# Understanding the structural degradation of South American historical silk: A Focal Plane Array (FPA) FTIR and multivariate analysis

**DOI:** 10.1038/s41598-019-53763-5

**Published:** 2019-11-21

**Authors:** Diego Badillo-Sanchez, David Chelazzi, Rodorico Giorgi, Alessandra Cincinelli, Piero Baglioni

**Affiliations:** Department of Chemistry “Ugo Schiff” and CSGI, University of Florence, Via della Lastruccia 3, 50019 Sesto Fiorentino, Florence Italy

**Keywords:** Chemistry, Analytical chemistry, Infrared spectroscopy

## Abstract

Silk artifacts constitute an invaluable heritage, and to preserve such patrimony it is necessary to correlate the degradation of silk fibroin with the presence of dyes, pollutants, manufacturing techniques, etc. Fourier Transform Infrared Spectroscopy with a Focal plane array detector (FPA FTIR) provides structural information at the micron scale. We characterized the distribution of secondary structures in silk fibers for a large set of South American historical textiles, coupling FTIR with multivariate statistical analysis to correlate the protein structure with the age of the samples and the presence of dyes. We found that the pressure applied during attenuated total reflectance (ATR) measurements might induce structural changes in the fibers, producing similar spectra for pristine and aged samples. Reflectance spectra were thus used for the rigorous characterization of secondary structures. Some correlation was highlighted between the age of the samples (spanning over five centuries) and specific changes in their secondary structure. A correlation was found between the color of the samples and structural alterations, in agreement with the chemical nature of the dyes. Overall, we demonstrated the efficacy of reflectance FPA µ-FTIR, combined with multivariate analysis, for the rigorous and non-invasive description of protein secondary structures on large sets of samples.

## Introduction

The term “silk” is generally used to indicate the product of the transformation of domesticated B*ombyx mori* cocoon’s spun fiber after the degumming process (i.e. a chemical treatment that removes the coating of sericin protein from the fiber^1^). Silk is mainly composed of Heavy-Fibroin, Light-Fibroin, and P25 (a chaperonin-like protein), in a 6:6:1 ratio^2^, distributed as amorphous and crystalline domains along the fiber axis^3,4^. Both the composition and structure of silk are responsible for its unique physical and mechanical properties, which justify its extended use in ancient fabrics and, more recently, in new bio-materials^5–10^. Historical silk artifacts constitute an invaluable heritage, usually present in museums and collections as clothes, tapestries, flags/banners and decorative objects. However, silk artifacts are often affected by degradation, mainly depending on environmental factors (relative humidity, light, temperature), and the presence of acids, oxidizing compounds, and biopollutants.

To preserve this cultural and historical patrimony, in-depth research into silk degradation mechanisms is a necessary step, prior to the design of cleaning and consolidation materials. Numerous methodologies to investigate the structure and dynamic behavior of silk are reported in the literature^11–17^. In particular, research aims to correlate the degradation of fibroin with the presence of dyes, fibers manufacturing techniques, pollutants, etc.^18–21^. Both Fourier Transform Infrared Spectroscopy (FTIR) and Raman have been extensively used to analyze proteins, thanks to the possibility of correlating spectral features with structural information^22–24^. The use of FTIR microscopy (µ-FTIR) in reflectance mode is advantageous as it allows the non-invasive analysis of non-transparent textile fibers. Since the amount of light reflected by some samples may be rather low, the reflectance spectra obtained with standard mercury cadmium telluride (MCT) detectors typically show low signal-to-noise (S/N) ratios^25,26^. Higher quality spectra are thus usually obtained by attenuated total reflectance measurements (ATR-FTIR), which guarantee higher S/N. In fact, many authors elected ATR as the standard technique for the investigation of textiles, including silk. For instance, this method has highlighted remarkable changes in the secondary structure of fibroin films induced by solvents or thermal treatment^27^; Belton *et al*. have shown the utility of ATR spectra fitting (without the use of peak deconvolution) to elucidate the conformational make up of silk protein in self-regenerated silk films^28^; however, in the case of naturally or artificially aged silk textiles, ATR analysis showed less evident changes in the secondary structure of aged fibroin compared to pristine silk. Vilaplana *et al*. reported that aged samples showed only a small increase in oxidation bands (1775–1700 cm^−1^), while no changes were observed in the Amide I region (1700–1600 cm^−1^)^29^. Smith *et al*. demonstrated the efficacy of ATR to identify silk in historical textiles from characteristic absorptions, however the ATR spectra of the aged silk samples showed no significant differences from those of reference pristine silk^30^. In addition, Koperska *et al*. used variations in the intensity ratio of Amide I, II and III bands in the ATR spectra of historical silk as estimators of crystallinity, oxidation, and depolymerization; while some relevant differences in the estimators were highlighted, the spectra of the aged and non-aged samples showed similar profiles in the Amide I-II region (1700–1450 cm^−1^)^31^. Garside *et al*. provided further insight by developing the use of polarized (Pol-) FTIR-ATR and near infrared (NIR) spectroscopy, and correlated the breaking strength of historical fibers with an orientated crystallinity parameter derived from the Pol-ATR spectra (i.e. comparing the ratio of β-sheet/α-helix absorption intensities of fibers aligned perpendicular and parallel to the incident electric vector)^32^. Nevertheless, no reproducible crystallinity index could be obtained through the more rigorous spectral deconvolution (peak fitting)^32^.

In this study, both reflectance and ATR measurements were carried out on pristine and historical silk fibers, comparing the results obtained through the systematic spectral deconvolution of the Amide I band. Measurements were performed using a Focal Plane Array (FPA) detector, consisting of a grid of light-sensitive elements, which allows acquiring simultaneously a large number of spectra on areas ranging from thousands of µm^2^ to tens of mm^2^, with high spatial resolution (e.g. 1.1–5.5 µm). Unless FPA detectors are used, such resolution is typically accessible only with synchrotron light^33^. FPA µ-FTIR 2D Imaging is an advantageous and feasible approach when investigating samples that have heterogeneous structure and composition at the micron-scale. Recently, we demonstrated the efficacy of this technique to study the distribution of secondary structures in silk fibers^34^. In particular, we showed that three samples with different age and color (e.g. fibers of flag banners from the 16^th^ and 19^th^ century) had reached different stages of a degradation process affecting the amorphous and crystalline domains of fibroin. However, no pattern was observed linking the age (or color) of the samples with the progress of the degradation process.

To fill this gap, in this work we investigated the correlation of the protein structure of a large set of historical silk samples with their age, and with the presence of dyes. Namely, FPA µ-FTIR was carried out on samples of historical flags and banners of different origins, obtained from the textile collection of the National Museum of Colombia, where the objects were kept in the last 200 years under identical conditions (in terms of light, relative humidity, temperature, etc.) and did not undergo any restoration intervention. First of all, the difference between the information obtained through ATR and reflectance measurements was critically evaluated. Then, the analysis of the complete set of samples was carried out in reflectance mode, followed by the spectral deconvolution of the Amide I band for each sample, so as to clarify the secondary structure of the fibroin at the micron-scale. Finally, principal components analysis (PCA) was performed to correlate the different structures with the age and color of the samples.

## Methods

### Silk samples

A textile of modern industrial production (used as a representative pristine silk standard) and 61 historical silk samples were analyzed (See Supplementary Information, SI Tables 1–3). The modern sample (hereinafter “Mod”) was a beige silk with a density of 2.57 g/cm^3^, obtained from a local market in Florence. The 61 historical samples (named “HS1-61”) came from the textile collection of the National Museum of Colombia. The age of these historical samples range from ca. 100 to 500 years. The majority of the samples are dyed blue, yellow or red; six samples exhibited different colors (orange, green, purple and black). None of the textiles has been restored or treated during storage and exhibition in the collections.

### Treatment of modern silk

In order to produce, through accelerated aging, observable changes in the secondary structure of fibroin induced by acid hydrolysis and photo-oxidation, the modern silk samples were treated as follows: 1 cm^2^ of Mod textile was immersed in 2 mL of a solution at pH 4 (Buffer Solution, pH 4, Potassium hydrogen phthalate-based, J.T. Baker Analyzed® Reagent, Mallinckrodt Baker, Inc., Phillipsburg, USA), and kept in darkness at room temperature for 24 hours. Then, the sample was rinsed using water purified by a Millipore system (resistivity > 18 MΩ cm), and dried at room temperature. This sample was labeled as “Mod1”. To investigate the effects of UV-Vis light, a Mod sample of 1 cm^2^ was placed in a UV-Vis chamber for 30 days, following a procedure reported elsewhere^34^. These illumination conditions are meant to accelerate the natural aging that would be experienced by objects on display in museums, where illuminations of 50–100 lux are typically used. The photo-aged sample was labeled as “Mod2”.

### Fourier Transform Infrared (FTIR) 2D imaging-chemical mapping

Individual fibers (length 5 mm) were manually separated from the original textile samples and analyzed (without any pre-treatment) with a Cary 670 FTIR spectrophotometer coupled to a Cary 620 FTIR microscope (Agilent Technologies). Measurements were carried out in ATR and reflectance mode (over a gold-plated reflective surface); background spectra were collected either in air (ATR) or directly on the gold-plated surface (reflectance). The FTIR settings were as follows: 512 scans for each acquisition, spectral resolution of 2 cm^−1^, open windows, and spectral range of 3900–900 cm^−1^. ATR data were collected using a Ge crystal, while for reflectance measurements a 15x Cassegrain objective was used., A Focal Plane Array (FPA) detector was employed for both ATR and reflectance measurements: for ATR, a 64 × 64 pixels grid was used (each pixel providing an independent spectrum related to an area of 1.1 × 1.1 µm^2^); for reflectance measurements, a 128 × 128 pixels grid was used (each pixel related to an area of 5.5 µm × 5.5 µm^2^). Each analysis produced a “tile” of 70 × 70 µm^2^ (ATR) or 700 × 700 µm^2^ (reflectance). In the FTIR 2D maps, the chromatic scale shows the intensity of bands, following the order red > yellow > green > blue.

### Analysis of the secondary protein structures

An IR map was collected (in ATR or reflectance mode) on a chosen spot for each silk fiber. Then, from each map, five spectra with high Amide A absorbance, and fifteen spectra of the air in contact with the Ge crystal (ATR), or of the golden platelet (reflectance), were selected. The spectra of air (or of the Au surface) were averaged to obtain a single spectrum, which was used as a reference to subtract environmental water absorptions from the fiber spectra. Then, each of the five fiber spectra underwent the following process: (1) Manual spectral subtraction of the reference air (or Au) spectrum; the subtraction factor was adjusted manually until no absorption at 1654 cm^−1^ (OH bending, H_2_O) was observable in the fiber spectra; (2) Smoothing with an SG quad-cubic function of 13–15 points, taking care not to alter any diagnostic feature of the spectra; (3) Spectrum truncation down to the 1720–1480 cm^−1^ range (Amide I–II region); (4) Baseline correction using a linear function connecting the two extremes of the truncated spectra; (5) Each spectrum was normalized to the maximum absorbance value of the Amide I band. Operations 1–5 were carried out using the Agilent Resolution Pro software (Agilent technologies). Each resultant spectrum was deconvoluted and fitted using the multipeak fitting package of the Igor Pro software, version 7 (WaveMetrics, Inc). First, the second derivative of the convoluted spectra was used to locate the position of bands. Then, the spectra were deconvoluted using Gaussian curves and a constant baseline (constrained at zero absorbance), in two steps: (1) The position and width of the bands were hold; the height of all bands was constrained to a maximum of 80 and a minimum of 0; the fitting was then iterated until no changes were reported between two successive iterations; (2) The width was let change in the 0–20 limit, and the height was let change with a minimum limit of 0; the fitting was iterated until no changes were reported between two successive iterations. The resultant deconvoluted bands composing Amide I were assigned to the different secondary structures of the protein as follows^35,36^: (Tyr) side chains/aggregated strands, 1605–1615 cm^−1^; aggregate β-strand/intermolecular β-sheets (weak), 1616–1621 cm^−1^; intermolecular β-sheets (strong), 1622–1627 cm^−1^; intramolecular β-sheets (strong), 1628–1637 cm^−1^; random coils/extended chains, 1638–1646 cm^−1^; random coils, 1647–1655 cm^−1^; α-helices, 1656–1662 cm^−1^; β-turns, 1663–1670, 1671–1685, and 1686–1696 cm^−1^; intermolecular β-sheets (weak), 1697–1703 cm^−1^; oxidation bands, 1703–1720 cm^−1^.

### Statistical data analysis

The secondary structures of the different silk samples, as obtained by the FTIR deconvolution/fitting process, were subjected to multivariate statistical analysis through Principal Component Analysis (PCA), using the Simca software Vs 16.0.1 (Sartorius stedim data analytics AB, Germany). PCA fundamentals on data treatment can be found elsewhere^37–42^. Data acquired on historical and aged samples were centered on the values obtained for pristine silk (“Mod” sample).

### Fiber Optics Reflectance Spectroscopy (FORS)

FORS analysis was performed on the silk fibers to identify dyes, using a portable optical fiber spectrophotometer PrimeTM X (B&W Tec Inc., Newark, DE, USA) with a back-thinned CCD array detector (1024 pixel), connected to a Deuterium/Tungsten light source and a bifurcated fiber reflectance probe that combines optical fibers (7 fibers, Ø = 200 μm each). Spectra were collected for each sample in the 200–900 nm interval, avoiding areas affected by noticeable defects; a 99% Teflon diffuse reflectance metrological standard from BW Tech (Newark, DE, USA) was employed for calibration. Each spectrum was collected averaging 50 cycles (50 ms each) to enhance S/N; both incident and acquisition angles are perpendicular to the surface. A dedicated software (BWSpec 3.27 by B&W Tec Inc.) was used for spectra acquisition and colorimetric data collection. The collected spectra were compared with those reported in the literature for the identification of dyes^43–47^.

## Results and Discussion

**Figure 1:**
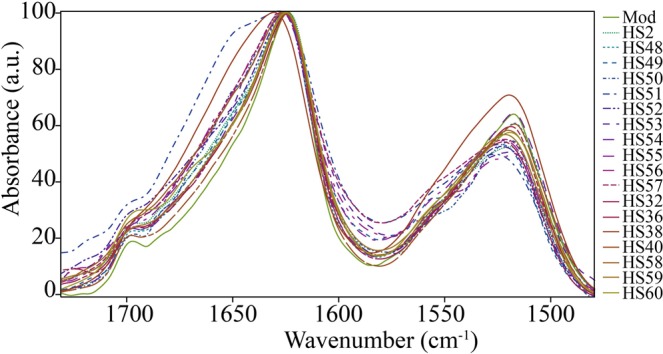
FPA ATR µ-FTIR spectra of pristine commercial silk (“Mod”), and of a subset of 18 historical silk samples, showing the Amide I and II region (1720–1480 cm^−1^). Each spectrum refers to a single pixel (1.1 × 1.1 µm^2^) from the 2D Imaging map of the corresponding sample (representative ATR maps of pristine and historical samples are shown in Figs. SI2 and SI4).

Figure 1 shows the ATR-FTIR spectra of pristine commercial silk (“Mod”), and of a set of 18 historical silk samples, in the 1720–1480 cm^−1^ range. This interval was highlighted, as it is used in the deconvolution/fitting process to evaluate the distribution of protein secondary structures. All the spectra exhibit the typical absorptions of B*ombyx mori* silk (as reported in the literature^29,48^) across the full Mid IR range explored by the FPA detector (3900–900 cm^−1^, see SI Figs. 2–4). Each spectrum in Fig. 1 refers to a single pixel (1.1 × 1.1 µm^2^) from the 2D Imaging map of the corresponding sample. The spectra were normalized to the maximum absorbance of Amide I, and a linear baseline was applied between 1720 and 1480 cm^−1^, prior to the deconvolution step. Normalization also facilitates the qualitative comparison of the spectral profiles. Almost all spectra exhibit Amide I and II absorptions that resemble those of non-aged *Bombyx mori* silk (“Mod”)^29^. In particular, the Amide I band is centered at 1625 cm^−1^, with only weak band components observable in some cases between 1640 and 1690 cm^−1^. The main exceptions are samples HS50 and HS49, respectively a blue (168 years) and a yellow (143 years) textile, which exhibit a broader band with a shoulder at 1647 cm^−1^.

**Figure 2:**
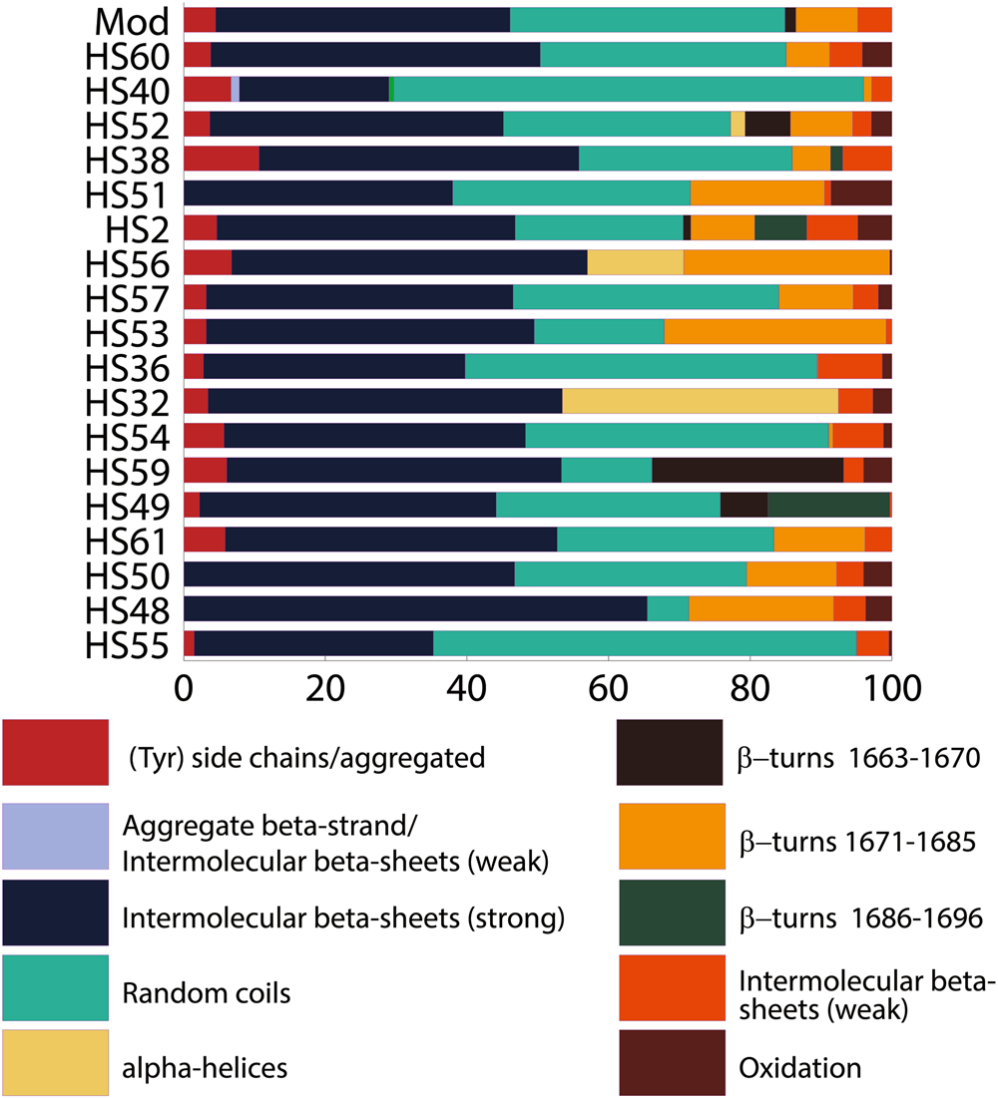
Assigned secondary structures (%) of silk protein, obtained from the deconvolution of the Amide I and II region (1720–1480 cm^−1^) of the FPA ATR µ-FTIR spectra of commercial (Mod) and historical silk (subset of 18 samples).

To get further insight, five spectra for each sample (related to five different pixels in each sample’s 2D Imaging map) were deconvoluted, and the average resulting structures are shown in Fig. 2. As a general trend, the proportion between β-sheets and random coils is close to 54:40, which is in agreement with the description (obtained via X-Ray Diffraction, XRD) of fibroin crystallites uniformly embedded in an amorphous matrix, commonly reported in the literature for standard *Bombyx mori* silk^27,29,36,49^. However, when the detailed composition of the secondary structure of different samples is critically analyzed, a striking feature emerges, i.e. the absence of several characteristic types of structures that are normally expected (with different proportions) both in pristine and historical samples. In particular, intramolecular β-sheet (strong) (1637–1628 cm^−1^), aggregate β-strand/intermolecular β-sheet (weak) (1621–1616 cm^−1^), and random coils/extended chains (1646–1638 cm^−1^) are completely missing in all samples. This is in strong disagreement with what we had previously observed in reflectance mode for the Mod sample and three historical samples (see also SI Figs. 1, 3 and 5 and Table 4), where these structures were observed^34^. While it might be possible that in some (but reasonably not all) historical samples these crystalline structures disappear as a result of late degradation stages^49^, such structures should be present at least for the pristine *Bombyx mori* silk^27^. Similar considerations apply to α-helices (1662–1656 cm^−1^) and two types of β-turns (1670–1663, and 1696–1686 cm^−1^), which are absent in the ATR spectra of the majority of the samples, including pristine silk. Moreover, other types of structures in the ATR spectra are either significantly overexpressed (intermolecular β-sheet (strong) at 1627–1622 cm^−1^, random coils at 1655–1647 cm^−1^, and side chains/aggregated strands at 1615–1605 cm^−1^) or underexpressed (intermolecular β-sheet (weak) at 1703–1697 cm^−1^) as opposed to reflectance measurements^34^. The proportion of β-turns (1685–1671 cm^−1^) and oxidation bands (1720–1703 cm^−1^) are instead in agreement with reflectance data. Overall, the deconvolution of ATR-FTIR spectra would suggest that almost all the considered historical samples have secondary structures similar to that of modern pristine silk, which is in contrast with the macroscopic evidence that all the historical samples in the set (61 specimens) exhibit significantly poorer mechanical properties than the Mod sample. In fact, it is well known that mechanical properties depend on the arrangement of secondary structures in silk fibers^50,51^.

Similarly, aging the Mod textile did not produce any significant change in the ATR spectra of the samples (see SI, Figs. 6 and 7), which again show profiles that match that of pristine silk, regardless of the aging protocol (i.e. using acid pH or UV-Vis photo-aging).

We hypothesized that the pressure applied during ATR measurements might induce structural changes that favor the formation of some motifs over others, producing similar profiles for both pristine and naturally (or artificially) aged samples. In fact, in order to obtain adequate band intensities, an intimate contact is required between the Ge crystal and the textile samples during the ATR experiments, and the inevitable pressure on the fibers might induce microstructural modifications^52^. This is reasonable considering that silk fibroin is a polymorphic material whose structure and structural dynamics are sensitive to experimental conditions such as pH, temperature, and pressure^35,53^. He at al. recently investigated the effects of low pressure on secondary structure transitions in wet films of regenerated silk fibroin^54^; these authors found that the application of low pressure in ATR experiments favors the formation of β-sheets, as seen by increasingly stronger absorptions in the ATR spectra around 1618 cm^−1^, analogously to what we observed here on silk fibers. Elsewhere, the conversion of silk III structure (threefold extended helix) to silk II (a β-sheet structure) owing to surface pressure on a Langmuir trough was reported^55^.

In conclusion, while ATR is typically considered as a standard tool for inquiring changes in commercial and historical textiles^29,36,56–58^, our results clearly show that, although in some cases partial changes in the spectra profile are observable, this technique is unsuitable when a rigorous characterization of silk secondary structure is needed, probably owing to the effects of the pressure applied during the measurements. Therefore, detailed information on the structural changes of the naturally and artificially aged silk samples was obtained through reflectance µ-FTIR.

**Figure 3:**
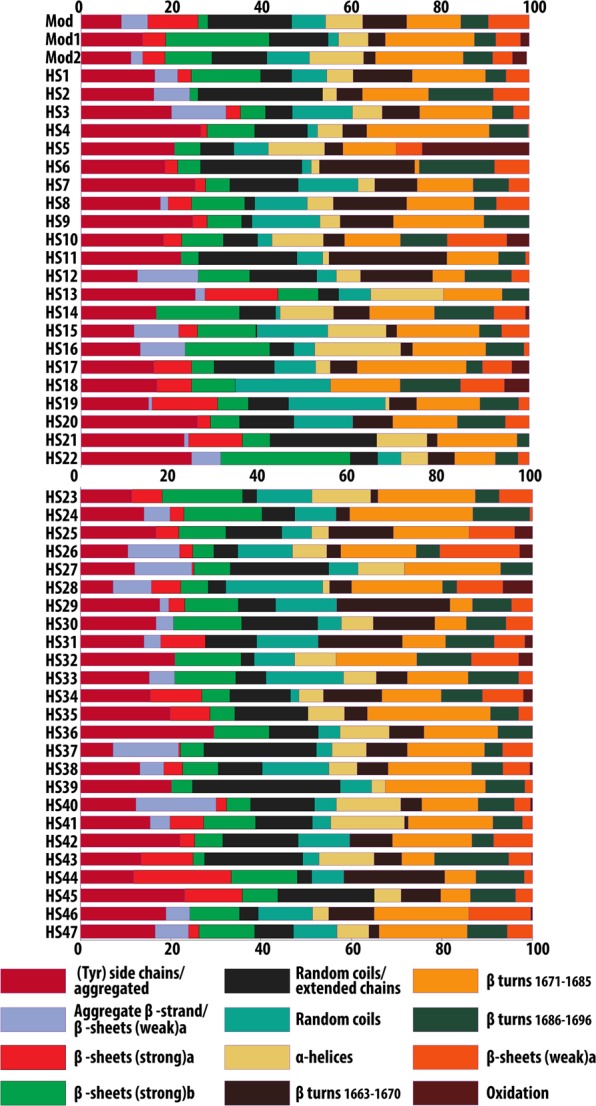
Assigned secondary structures (%) of silk protein, obtained from the deconvolution of the Amide I and II region (1720–1480 cm^−1^) of reflectance FPA µ-FTIR spectra of commercial (Mod, Mod1-2) and historical silk samples (full set, HS1-47).

The distribution of the secondary structures for a set of 50 samples (Mod, Mod1-2, HS1-47), obtained by reflectance µ-FTIR, is shown in Fig. 3 (see also SI Table 5). A heterogeneous distribution of structures is observed (as opposed to ATR measurements), which was reasonably expected for samples of various origins, age, content (e.g. dyes), and that were likely exposed to different manufacturing processes. Artificial aging of pristine silk at pH 4 (Mod1) produced a strong decrease of aggregate β-strand/intermolecular β-sheets (weak), intermolecular β-sheets (strong and weak), random coils/extended chains, and random coils; an increase in (Tyr) side chains/aggregate, intramolecular β-sheets (strong), β-turns (1685–1671 cm^−1^), and oxidation bands was also observed. This is consistent with the hydrolysis of amorphous phases, and the consequent formation of smaller, more isolated crystalline domains^36,59^. UV-Vis aging (Mod2) produced a decrease of aggregate β-strand/intermolecular β-sheets (weak), intermolecular β-sheets (strong and weak), random coils/extended chains, and β-turns (1670–1663 cm^−1^), and an increase of (Tyr) side chains/aggregate, intramolecular β-sheets (strong), β-turns (1685–1671 cm^−1^), random coils, α-helices, and oxidation bands; this result is consistent with the oxidation of side chains and amorphous domains following irradiation, as reported in the literature^49,59,60^.

In order to inquire the presence of possible correlations among the secondary structures of the samples, we analyzed the experimental data through a PCA model, an approach that has been extensively used to classify calculated structures in proteins^61^, identify spectroscopic markers^29,48,62^, and determine protein dynamics^63^. The model uses as variables the percentage amount of the structures obtained by the deconvolution/fitting of the µ-FTIR reflectance spectra (all the 12 different secondary structures), plus the samples age, for 50 silk samples (Mod, Mod1-2, HS1-47). The observations were centered around the values of pristine silk (Mod) so as to highlight differences in the historical samples with respect to non-aged silk. The PCA model yields a cumulative R^2^ of 0.78 (R1 = 0.67; R2 = 0.11) and a Q^2^ of 0.40 (Q1 = 0.43; Q2 = 0.40) for the first two principal components. These results were deemed as acceptable, considering the high variability in the secondary structure exhibited by historical samples with different provenance, aging, and storing conditions prior to their inclusion in a monitored collection.

**Figure 4:**
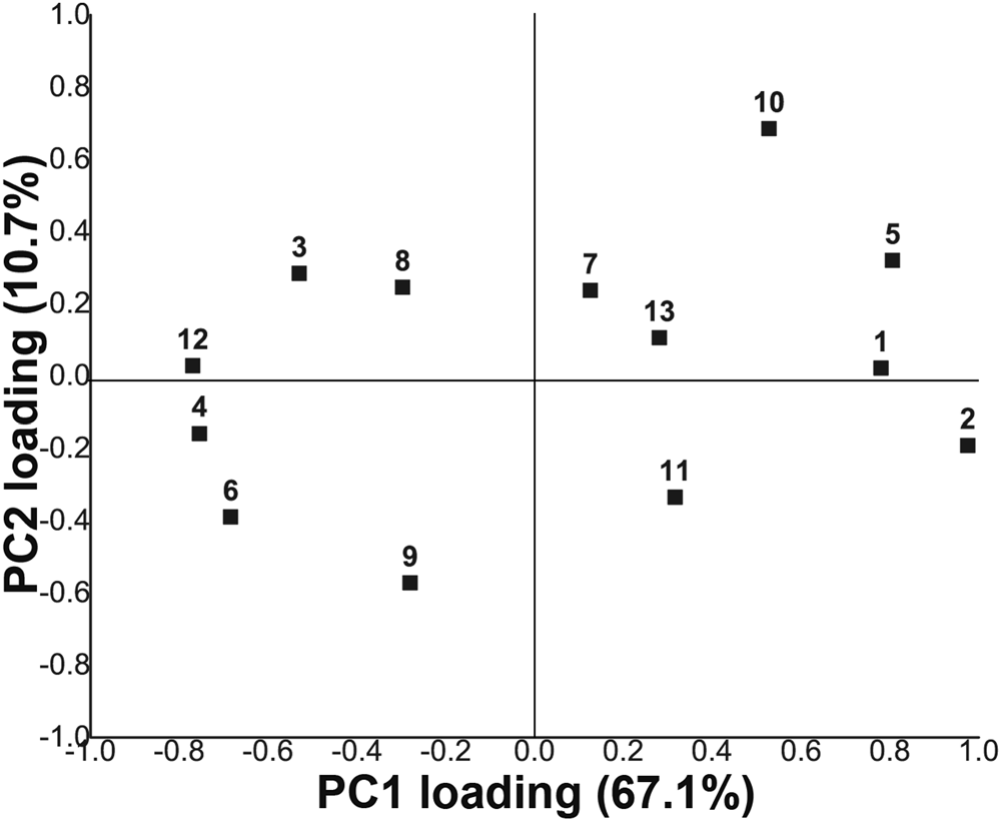
Loading plot (first two principal components, PC1,2) of the PCA model applied to the secondary structures and age of a set of 50 silk samples (Mod, Mod1-2 and HS1-47). The variables are numbered in the plot as follows: age (1), (Tyr) Side chains/aggregated β-strands (2), aggregate β-strand/β-sheets (weak) (3), intermolecular β-sheets (strong) (4), intramolecular β-sheets (strong) (5), random coils/extended chains (6), random coils (7), α-helices (8), β-turns (1663–1670 cm^−1^) (9), β-turns (1671–1685 cm^−1^) (10), β-turns (1686–1696 cm^−1^) (11), intermolecular β-sheets (weak) (12), oxidation (13).

The loading plot (see Fig. 4) of the first two principal components (PC1,2), shows that PC1 mainly accounts for the variance of crystalline structures, while variance of amorphous structures is predominant in PC2. Positive contributions to PC1 mainly come from (Tyr) side chains/aggregated strands, intramolecular β-sheets (strong), and β-turns (1671–1685 cm^−1^), along with the age of the samples; oxidation bands provide a smaller contribution. Aggregate β-strand/intermolecular β-sheets (weak), intermolecular β-sheets (strong), random coils/extended chains, and to a lesser extent α-helices, provide negative contributions. For the considered set of historical silk samples, age can be thus correlated with the degradation of amorphous regions, which produces smaller and more isolated crystalline domains. These findings are in good agreement with the literature, where the degradation of fibroin is reported by some authors to start at the amorphous domains, progressively affecting crystalline regions and leading to loss of alignment of β-sheets, until short range order is lost at the latest stage, accompanied by chain scission and strong decrease of crystallinity^29,36,49,59^. However, it is also evident from the model that age cannot be univocally considered as the sole parameter to evaluate changes in the secondary structure. This observation is supported by archeological findings showing that older silk artifacts do not necessarily exhibit worse conservation status than more recent objects^32,45,58,64–68^. Other factors are thus expected to play significant roles in determining changes of the silk protein.

**Figure 5:**
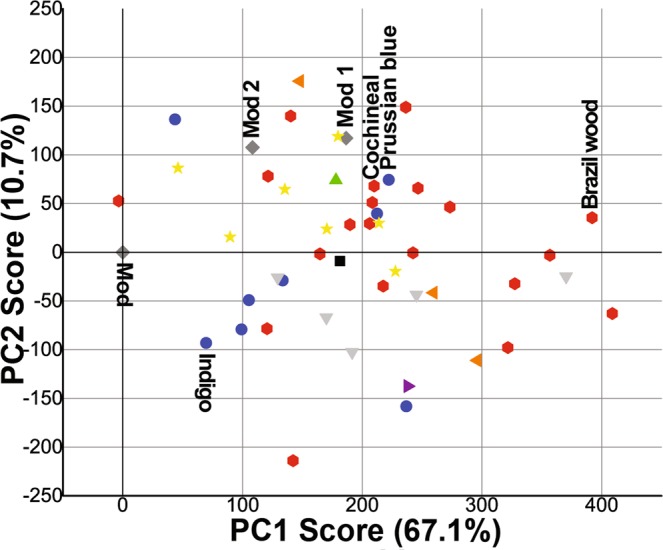
Score plot for PC1 and PC2 of the PCA applied to the different secondary structures and age for a set of 50 silk samples (Mod, Mod1-2 and HS1-47). In the plot, each silk sample is associated with its color, or marked as gray if non-dyed. Names of the main dyes are specified for each portion of the plot.

The score plot obtained from PC1 and PC2 shows a distribution of the samples, with pristine silk (Mod) at the origin of axes, and historical (HS1-47) and artificially aged (Mod1-2) samples shifted to positive values of PC1 (*x* axis) and positive or negative values of PC2 (*y* axis). When only the position of the samples in the plot is considered (see SI, Fig. 8), no major groups can be distinguished among the silk samples. However, when each sample is associated to its color (Fig. 5), some groups and trends are clearly highlighted. Namely, red samples tend to be located at farther distances from Mod, with large positive values of PC1, i.e. their secondary structure is the most dissimilar from pristine fibers, with the highest increase of intramolecular β-sheets (strong) and (Tyr) side chains/aggregated strands, and some increase of oxidation bands. Blue and yellow samples are in general grouped closer to Mod, roughly halfway along the PC1 axis, but blue samples tend to have negative values of PC2 (increase of random coils/extended chains), while yellow samples have mostly positive PC2 values (increase of α-helices, random coils, and oxidation bands). The yellow samples show also a tighter grouping around Mod1-2. It must be noticed that samples within the same color group have different ages.

The identification of dyes in the historical samples, using FORS (see SI, Table 6 and Figs. 9–12), allowed further explanation of the observed trends.

The red dyes were found to be cochineal, brazilwood, and madder (see SI, Fig. 9). These dyes contain polycyclic aromatic groups, including anthraquinone structures, which are able to form complexes with proteins and metal ions used in the dying treatment (e.g. Al ions^69^). Such complexes include extended conjugated structures able to promote the photo-oxidation and weakening of silk proteins; in fact, anthraquinone derivatives are known to be protein photocleavers^70^. Moreover, anthraquinoid dyes are in some cases applied at acidic pH values, in order to achieve the desired hue and stability^69^. These features are consistent with the fact that the red dyed historical fibers have secondary structures characterized by smaller and less interconnected crystalline domains, decreased amorphous content, and more oxidized groups than pristine silk. For some of the red samples (e.g. Brazil wood, see Fig. 5) this trend is more pronounced, while others (e.g. Cochineal) are relatively less degraded and closer in structure to Mod1-2.

Blue dyes were identified as Prussian Blue and Indigo (see SI, Fig. 10). Overall, the blue samples exhibit secondary structures that resemble those of pristine silk more than red samples, even though some (including one Prussian Blue) are also close to Mod1-2. In general, blue samples exhibit some increase of (Tyr)side-chains/aggregated strands, random coil/extended chains and β-turns (1696–1686 cm^−1^). This indicates that slight degradation of the crystalline domains has occurred, leading to an increase of amorphous structures, and to the exposure, rather than scission, of side chains. Macroscopically, the blue dyed fibers show better mechanical behavior than red and yellow samples. In fact, Indigo does not form complexes with the silk protein groups, as opposed to anthraquinoids; instead, the dye-protein interaction is based on ionic and secondary bonds (hydrogen bonds, polar forces and Van der Waals interactions)^71^. The absence of extended conjugated structures is thus expected to limit the photo-oxidation (and consequent degradation) of the indigo dyed fibers.

Most of the yellow dyed samples are grouped around Mod1 and Mod2. Overall, these fibers show secondary structures with decreased intermolecular β-sheets, and increased oxidation groups and random coils, which is consistent with exposure to acidity and photo-oxidative aging^29^. Even though it was not possible to unequivocally identify the yellow dyes by FORS, their spectra resemble those of some natural dyes commonly and historically used, such as hydroxy and methoxy derivatives of flavones and isoflavones, dihydropyrans, anthocyananidins and carotenoids (see SI, Fig. 11). These dyes are typically linked to the fibers by the addition of a mordant, in most cases a metal salt whose cation bridges to the fibers through complexation^72^. Even in this case, strong absorption of Vis (blue) and UV light by the complexes can result in oxidation of the fibers. Black silk samples have also some proximity with Mod 1-2, suggesting that degradation might have occurred also through thermal transformation of oxidized products over prolonged exposition to light and thermal oscillations.

Samples with secondary colors (green, orange, purple) exhibit secondary structures with features ascribable to the presence of the different dyes mixed in their manufacture. Green samples (combination of blue and yellow dyes) show features and degradation patterns similar to those of yellow samples; orange samples (combination of red and yellow dyes) and purple samples (combination of red and blue dyes) followed degradation paths similar to those of red and blue samples, respectively.

## Conclusions

A large set of historical and pristine silk samples was analyzed through FPA µ-FTIR, comparing the information obtained from ATR and reflectance measurements. Even though the ATR spectra of the historical samples exhibit some differences from those of pristine silk, the rigorous deconvolution of Amide I absorptions showed the absence of characteristic types of structures that are expected both in pristine and historical silk. In contrast, the deconvolution of reflectance spectra showed a heterogeneous distribution of structures, consistent with the typical degradation paths that silk textiles undergo during natural aging. We concluded that the pressure applied during ATR measurements might be enough to induce structural changes in the silk fibers, favoring the formation of some structural motifs (e.g. intermolecular β-sheet strong) over others, and producing similar spectral profiles for pristine and aged samples.

The structures obtained by reflectance measurements for the full set of samples were then used as variables in PCA multivariate analysis aimed to investigate possible correlations among the structures of the silk textiles. Our results show that there is a correlation between the age of the historical samples (spanning over five centuries) and specific changes in their secondary structure. However, PCA results suggest that aging time cannot be univocally considered as the sole parameter in silk degradation. A correlation was found between the color of the samples and alterations of structures. In particular, silk samples containing red (anthraquinoids) and yellow (flavonoids) dyes exhibit secondary structures similar to those of acid- or photo-aged silk, and several red samples show even more pronounced degradation features along this trend. This is consistent with the formation of complexes between the dyes, metal cations (mordants) and the proteins, producing large conjugated structures that favor the photo-oxidative degradation of the fibers. Silk containing blue (indigo) dyes show less significant structural alterations, which was explained considering that indigo binds to proteins mostly through ionic and secondary interactions, without the formation of complexes with extended conjugation.

Overall, we demonstrated the efficacy of FPA µ-FTIR in reflectance mode, combined with multivariate analysis, for the rigorous and non-invasive description of protein secondary structures of large sets of samples. The developed approach constitutes a feasible analytical protocol for the investigation of structural changes in polymeric materials, with potential applications in several fields including cultural heritage preservation (diagnostics), forensics, material science, industrial quality control, and pharmaceutics.

## Supplementary information

Supplementary Information

## References

[1] Vyas SK, Shukla SR (2016). Comparative study of degumming of silk varieties by different techniques. The Journal of The Textile Institute.

[2] Inoue S (2000). Silk Fibroin of Bombyx mori Is Secreted, Assembling a High Molecular Mass Elementary Unit Consisting of H-chain, L-chain, and P25, with a 6:6:1 Molar Ratio. J. Biol. Chem..

[3] Wojcieszak M (2014). Origin of the variability of the mechanical properties of silk fibers: 4. Order/crystallinity along silkworm and spider fibers. Journal of Raman Spectroscopy.

[4] Vollrath, F., Porter, D. & Dicko, C. 5 - The structure of silk. In *Handbook of Textile Fibre Structure* **2**, 146–198 (Woodhead Publishing, 2009).

[5] Kaplan, D., Adams, W. W., Farmer, B. & Viney, C. (Eds) *Silk Polymers. Materials Science and Biotechnology* (American Chemical Society, 1994).

[6] Weber, R. S. Craig (auth.), C. L., Asakura, T. & Miller (eds), T. *Biotechnology of Silk*. (Springer Netherlands, 2014).

[7] Hu X (2013). Stability of Silk and Collagen Protein Materials in Space. Scientific Reports.

[8] Jiang P (2014). Spider silk gut: Development and characterization of a novel strong spider silk fiber. Scientific Reports.

[9] Rockwood Danielle N, Preda Rucsanda C, Yücel Tuna, Wang Xiaoqin, Lovett Michael L, Kaplan David L (2011). Materials fabrication from Bombyx mori silk fibroin. Nature Protocols.

[10] Min, K., Kim, S. & Kim, S. Silk protein nanofibers for highly efficient, eco-friendly, optically translucent, and multifunctional air filters. *Sci Rep* **8** (2018). 10.1038/s41598-018-27917-w PMC6018553 29941979

[11] Darshan GH, Kong D, Gautrot J, Vootla S (2017). Physico-chemical characterization of Antheraea mylitta silk mats for wound healing applications. Scientific Reports.

[12] Yarger JL, Cherry BR, van der Vaart A (2018). Uncovering the structure–function relationship in spider silk. *Nature Reviews*. Materials.

[13] Pérez-Rigueiro J (2019). Emergence of supercontraction in regenerated silkworm (Bombyx mori) silk fibers. Scientific Reports.

[14] Qin N (2016). Nanoscale probing of electron-regulated structural transitions in silk proteins by near-field IR imaging and nano-spectroscopy. Nature Communications.

[15] Vollrath F, Hawkins N, Porter D, Holland C, Boulet-Audet M (2014). Differential Scanning Fluorimetry provides high throughput data on silk protein transitions. Scientific Reports.

[16] Koski KJ, Akhenblit P, McKiernan K, Yarger JL (2013). Non-invasive determination of the complete elastic moduli of spider silks. Nature Materials.

[17] Lepore E, Isaia M, Mammola S, Pugno N (2016). The effect of ageing on the mechanical properties of the silk of the bridge spider *Larinioides cornutus* (Clerck, 1757). Scientific Reports.

[18] Aguayo T, Carolina Araya M, Mónica Icaza T, Campos-Vallette M (2014). A vibrational approach for the study of historical weighted and dyed silks. Journal of Molecular Structure.

[19] Das D, Datta DB, Bhattacharya P (2014). Simultaneous Dyeing and Finishing of Silk Fabric With Natural Color and Itaconic Acid. Clothing and Textiles Research Journal.

[20] Degano I, Biesaga M, Colombini MP, Trojanowicz M (2011). Historical and archaeological textiles: An insight on degradation products of wool and silk yarns. Journal of Chromatography A.

[21] Egerton GS (1948). Some Aspects of the Photochemical Degradation of Nylon, Silk, and Viscose Rayon. Textile Research Journal.

[22] Barth A (2007). Infrared spectroscopy of proteins. Biochimica et Biophysica Acta (BBA) - Bioenergetics.

[23] Arrondo JLR, Muga A, Castresana J, Goñi FM (1993). Quantitative studies of the structure of proteins in solution by fourier-transform infrared spectroscopy. Progress in Biophysics and Molecular Biology.

[24] Percot A (2014). Water dependent structural changes of silk from Bombyx mori gland to fibre as evidenced by Raman and IR spectroscopies. Vibrational Spectroscopy.

[25] Chalmers JM, Everall NJ, Ellison S (1996). Specular reflectance: A convenient tool for polymer characterization by FTIR-microscopy?. Micron.

[26] Fringeli U.P. (2017). ATR and Reflectance IR Spectroscopy, Applications. Encyclopedia of Spectroscopy and Spectrometry.

[27] Hu X, Kaplan D, Cebe P (2006). Determining Beta-Sheet Crystallinity in Fibrous Proteins by Thermal Analysis and Infrared Spectroscopy. Macromolecules.

[28] Belton DJ, Plowright R, Kaplan DL, Perry CC (2018). A robust spectroscopic method for the determination of protein conformational composition – Application to the annealing of silk. Acta Biomaterialia.

[29] Vilaplana F, Nilsson J, Sommer DVP, Karlsson S (2014). Analytical markers for silk degradation: comparing historic silk and silk artificially aged in different environments. Anal Bioanal Chem.

[30] Smith MJ, Thompson K, Hermens E (2016). Breaking down banners: analytical approaches to determining the materials of painted banners. Heritage Science.

[31] Koperska MA (2014). Degradation markers of fibroin in silk through infrared spectroscopy. Polymer Degradation and Stability.

[32] Garside P, Lahlil S, Wyeth P (2005). Characterization of historic silk by polarized attenuated total reflectance Fourier transform infrared spectroscopy for informed conservation. Appl Spectrosc.

[33] Ryu M (2017). Orientational Mapping Augmented Sub-Wavelength Hyper-Spectral Imaging of Silk. Scientific Reports.

[34] Badillo-Sanchez D, Chelazzi D, Giorgi R, Cincinelli A, Baglioni P (2018). Characterization of the secondary structure of degummed Bombyx mori silk in modern and historical samples. Polymer Degradation and Stability.

[35] Kong J, Yu S (2007). Fourier Transform Infrared Spectroscopic Analysis of Protein Secondary Structures. Acta Biochimica et Biophysica Sinica.

[36] Garside P, Wyeth P (2007). Crystallinity and degradation of silk: correlations between analytical signatures and physical condition on ageing. Appl. Phys. A.

[37] Martens H, Næs T (1984). Multivariate calibration. I. Concepts and distinctions. TrAC Trends in Analytical Chemistry.

[38] Næs T, Martens H (1984). Multivariate calibration. II. Chemometric methods. TrAC Trends in Analytical Chemistry.

[39] Slutsky, B. Handbook of Chemometrics and Qualimetrics: Part A By D. L. Massart, B. G. M. Vandeginste, L. M. C. Buydens, S. De Jong, P. J. Lewi, and J. Smeyers-Verbeke. Data Handling in Science and Technology Volume 20A. Elsevier: Amsterdam. 1997. Xvii + 867 pp. ISBN 0-444-89724-0. $293.25. *J. Chem. Inf. Comput. Sci*. **38**, 1254–1254 (1998).

[40] Ratola N, Amigo JM, Alves A (2010). Comprehensive assessment of pine needles as bioindicators of PAHs using multivariate analysis. The importance of temporal trends. Chemosphere.

[41] Lee LC, Liong C-Y, Jemain AA (2017). A contemporary review on Data Preprocessing (DP) practice strategy in ATR-FTIR spectrum. Chemometrics and Intelligent Laboratory Systems.

[42] Bereton, R. G. Pattern Recognition. in *Applied Chemometrics for Scientists* 145–191, 10.1002/9780470057780.ch5 (John Wiley & Sons, Ltd, 2007).

[43] Maynez-Rojas MA, Casanova-González E, Ruvalcaba-Sil JL (2017). Identification of natural red and purple dyes on textiles by Fiber-optics Reflectance Spectroscopy. Spectrochimica Acta Part A: Molecular and Biomolecular Spectroscopy.

[44] Angelini LG (2010). Characterization of Traditional Dyes of the Mediterranean Area by Non-Invasive Uv-Vis-Nir Reflectance Spectroscopy. Studies in Conservation.

[45] Gulmini M (2013). Identification of dyestuffs in historical textiles: Strong and weak points of a non-invasive approach. Dyes and Pigments.

[46] Acquaviva S, D’Anna E, De Giorgi ML, Della Patria A, Baraldi P (2010). Physical and chemical investigations on natural dyes. Appl. Phys. A.

[47] Leona M, Winter J (2001). Fiber Optics Reflectance Spectroscopy: A Unique Tool for the Investigation of Japanese Paintings. Studies in Conservation.

[48] Peets P, Leito I, Pelt J, Vahur S (2017). Identification and classification of textile fibres using ATR-FT-IR spectroscopy with chemometric methods. Spectrochimica Acta Part A: Molecular and Biomolecular Spectroscopy.

[49] Li M-Y (2013). Study of the degradation mechanism of Chinese historic silk (Bombyx mori) for the purpose of conservation. Polymer Degradation and Stability.

[50] Colomban P, Dinh HM, Bunsell A, Mauchamp B (2012). Origin of the variability of the mechanical properties of silk fibres: 1 - The relationship between disorder, hydration and stress/strain behaviour. Journal of Raman Spectroscopy.

[51] Jauzein, V. & Colomban, P. 6 - Types, structure and mechanical properties of silk. in *Handbook of Tensile Properties of Textile and Technical Fibres* (ed. Bunsell, A. R.) 144–178 (Woodhead Publishing, 2009).

[52] Van Nimmen E (2008). FT-IR spectroscopy of spider and silkworm silks: Part I. Different sampling techniques. Vibrational Spectroscopy.

[53] Wilson D, Valluzzi R, Kaplan D (2000). Conformational Transitions in Model Silk Peptides. Biophysical Journal.

[54] He Z, Liu Z, Zhou X, Huang H (2018). Low pressure-induced secondary structure transitions of regenerated silk fibroin in its wet film studied by time-resolved infrared spectroscopy. Proteins.

[55] Valluzzi R, Gido SP, Zhang W, Muller WS, Kaplan DL (1996). Trigonal Crystal Structure of Bombyx mori Silk Incorporating a Threefold Helical Chain Conformation Found at the Air−Water Interface. Macromolecules.

[56] Huang D (2013). A new consolidation system for aged silk fabrics: Effect of reactive epoxide-ethylene glycol diglycidyl ether. Reactive and Functional Polymers.

[57] Liu J (2011). Identification of ancient textiles from Yingpan, Xinjiang, by multiple analytical techniques. Journal of Archaeological Science.

[58] Nilsson J, Vilaplana F, Karlsson S, Bjurman J, Iversen T (2010). The Validation of Artificial Ageing Methods for Silk Textiles Using Markers for Chemical and Physical Properties of Seventeenth-Century Silk. Studies in Conservation.

[59] Hirabayashi K, Yanagi Y, Kawakami S, Okuyama K, Hu W (1987). Degradation of silk fibroin. The Journal of Sericultural Science of Japan.

[60] Lu Q (2011). Degradation Mechanism and Control of Silk Fibroin. Biomacromolecules.

[61] Howe PWA (2001). Principal components analysis of protein structure ensembles calculated using NMR data. J Biomol NMR.

[62] Yu X (2018). Surface enhanced Raman spectroscopy distinguishes amyloid Β-protein isoforms and conformational states. Protein Science.

[63] David CC, Jacobs DJ (2014). Principal component analysis: a method for determining the essential dynamics of proteins. Methods Mol. Biol..

[64] Liu M (2015). Identification of Ancient Silk Using an Enzyme-linked Immunosorbent Assay and Immuno-fluorescence Microscopy. Analytical Sciences.

[65] Zhang X, Wyeth P (2010). Using FTIR spectroscopy to detect sericin on historic silk. Sci. China Chem..

[66] Barrigón M (2015). Textiles and Farewells: Revisiting the Grave Goods of King Alfonso VIII of Castile and Queen Eleanor Plantagenet. Textile History.

[67] Karl B (2014). Silk and Propaganda — Two Ottoman Silk Flags and the Relief of Vienna, 1683. Textile History.

[68] Levey SM (1969). Illustrations of the History of Knitting Selected from the Collection of the Victoria and Albert Museum. Textile History.

[69] Bechtold, T. Natural Colorants – Quinoid, Naphthoquinoid and Anthraquinoid Dyes. in *Handbook of Natural Colorants* (eds Bechtold, T. & Mussak, R.) 151–182 (John Wiley & Sons, Ltd, 2009).

[70] Suzuki A, Hasegawa M, Ishii M, Matsumura S, Toshima K (2005). Anthraquinone derivatives as a new family of protein photocleavers. Bioorganic & Medicinal Chemistry Letters.

[71] Steingruber, E. Indigo and Indigo Colorants. In *Ullmann’s Encyclopedia of Industrial Chemistry* (Wiley-VCH Verlag GmbH & Co. KGaA, 2000).

[72] Rosenberg E (2008). Characterisation of historical organic dyestuffs by liquid chromatography–mass spectrometry. Anal Bioanal Chem.

