# Supplementary material for: Understanding the structural degradation of South American historical silk: A Focal Plane Array (FPA) FTIR and multivariate analysis
Source: Sci Rep. 2019 Nov 21;9:17239. doi: 10.1038/s41598-019-53763-5 (PMC6872790; doi:10.1038/s41598-019-53763-5)
Supplement: Supplementary file 1 — Supplementary Information [file 41598_2019_53763_MOESM1_ESM.docx]

Supplementary Information

Understanding the structural degradation of South American historical silk: A Focal Plane Array (FPA) FTIR and multivariate analysis

Diego Badillo-Sanchez ^a,b^‡^*^, David Chelazzi ^b^‡^*^, Rodorico Giorgi^b^, Alessandra Cincinelli ^a^, Piero Baglioni ^b^

^a^Department of Chemistry “Ugo Schiff” ^b^University of Florence and CSGI-Florence. Via della Lastruccia 3, 50019, Sesto Fiorentino (Florence, Italy)

SI Table 1. List of Flags (belonging to the National Museum of Colombia), from which silk samples were collected and analyzed in this study

| Museum ID flag reference | Name | Possible provenance | Date (year) | Dimensions (cm) |
| --- | --- | --- | --- | --- |
| 97 | Coat of arms of the royal audience of Santa Fe | Spain | Ca. 1550 | 164 x 133 x 5 |
| 99 | Flag with the coat of arms of Castilla y León | Spain | Ca. 1700 | 159 x 155 |
| 100 | Flag of the infantry regiment of "los Cazadores de Extremadura, second battalion" | Spain | Ca. 1815 | 158 x 150 |
| 101 | Flag of the Spanish regiment "of Burgos" | Spain | Ca. 1815 | 145 x 143 |
| 103 | Flag of a Spanish battalion of infantry | Spain | Ca. 1813 | 150 x 140 |
| 104 | Flag of the Numancia battalion | Spain | Ca. 1813 | 148 x 145 |
| 105 | Gran Colombia's flag from the Hussars' battalion | Colombia | Ca. 1824 | 73 x 78 |
| 106 | Gran Colombia's flag from the Hussars del centro' battalion | Colombia | Ca. 1824 | 79 x 88 |
| 107 | Gran Colombia's flag from the Milicias Regladas de Cartagena' battalion | Colombia | Ca. 1823 | 168 x 162,5 |
| 108 | Flag of the first military auxiliary battalion of the province of Bogota | Colombia | Ca. 1824 | 153 x 153 |
| 109 | Flag with the coat of arms of King Carlos IV of Spain | Spain | Ca. 1790 | 104,5 x 160 |
| 110 | Gran Colombia's flag of the Ligeros No1' battalion | Spain | Ca. 1824 | 160,7 x 153 |
| 111 | Gran Colombia's flag of the National Artillery Brigade, of Cundinamarca | Colombia | Ca. 1824 | 143 x 195 |
| 113 | Flag from one of the Colombian civil wars | Colombia | Ca. 1850 | 147,8 x 166 |
| 114 | "Estados Unidos de Colombia's Flag", dedicated to the Cauca´s liberators | Colombia | Ca. 1861 | 255 x 155 |
| 115 | Banner taken at the Panama Chagres capitulation | Spain | Ca. 1815 | 98 x 124 |
| 116 | Flag with the coat of arms of Castilla y León from the "Escuadrón de Vaqueanos del Batallón General" | Spain | Ca. 1700 | 74 x 106 |
| 117 | Banner with the Spanish coat of arms | Spain | Ca. 1808 | 168 x 162,5 |
| 118 | Banner with the Castilla y León coat of arms | Spain | Ca. 1808 | 134 x 111 |
| 120 | Venezuelan flag taken at the Garrapecera battle | Venezuela | 1901 | 144 x 185 |
| 122 | Gran Colombia's flag from the Simón Bolívar No. 17 battalion | Colombia | 1824 | 178,5 x 253 |
| 124 | Arequipa's coat of arms | Peru | Ca. 1875 | 107 x 80 |
| 126 | Colombian Sovereignty flag which sailed over the amazon river after the Salomón Lozano frontiers treaty | Colombia | 1929 | 65 x 95,5 |
| 129 | Colombian flag gifted to the Gr. Manuel Briceño | Colombia | 1885 | 247 x 233,5 |
| 1948 | Estados unidos de Colombia's coat of arms | Venezuela | s. XIX | 173 x 183 |
| 3044 | Colombian flag that owns to José Hilario López | Colombia | Ca. 1849 | 280 x 195 |
| 3245 | Banner that owns to Gr. Pedro Alcántara Herrán | Colombia | s. XIX | 49 x 54,3 |
| 3305 | Estados Unidos de Colombia's coat of arms from the Chía No. 27 army battalion | Spain | Ca. 1538 | 105 x 34 |
| 3615 | Battalion Rifles No. 14 of the republic of Colombia coat of arms | Colombia | Ca. 1899 | 81,2 x 64,8 |
| 6079 | Colombian flag that owns to the Gr. Solón Wilches | Colombia | Ca. 1880 | 42 x 23 |
| 7354 | Battalion Santos N° 4 flag won in the Palonegro battle for the Gr. Josué Calasanz Guevara | Colombia | 1900 | 173 x 183 |

SI Table 2. List of silk samples used for ATR µ-FTIR analysis

| Sample | Date | Museum ID flag |
| --- | --- | --- |
| Mod | 2015 | -- |
| HS2 | 1901 | 120 |
| HS48 | 1880 | 6079 |
| HS49 | 1875 | 124 |
| HS50 | 1850 | 113 |
| HS51 | 1901 | 1948 |
| HS52 | 1860 | 3245 |
| HS53 | 1824 | 108 |
| HS54 | 1824 | 106 |
| HS55 | 1823 | 107 |
| HS56 | 1815 | 115 |
| HS57 | 1813 | 103 |
| HS32 | 1813 | 104 |
| HS36 | 1808 | 118 |
| HS38 | 1790 | 109 |
| HS40 | 1700 | 116 |
| HS58 | 1700 | 99 |
| HS59 | 1700 | 99 |
| HS60 | 1550 | 97 |
| HS61 | 1538 | 3305 |

SI Table 3. List of silk samples used for reflectance µ-FTIR analysis. MNC: Fiber not colored, originally located inside a metal thread in the artifact; NC: Not colored; --: Not measured

| Sample | Date | Museum ID flag | Color | Age |
| --- | --- | --- | --- | --- |
| Mod | 2015 | MOD | NC | 3 |
| Mod1 | 2018 | Mod pH 4 | NC | 1 |
| Mod2 | 2018 | Mod UV | NC | 1 |
| HS1 | 1929 | 126 | Red | 89 |
| HS2 | 1901 | 120 | Red | 117 |
| HS3 | 1901 | 1948 | Red | 117 |
| HS4 | 1900 | 7354 | Yellow | 118 |
| HS5 | 1900 | 7354 | Red | 118 |
| HS6 | 1899 | 3615 | Red | 119 |
| HS7 | 1885 | 129 | Yellow | 133 |
| HS8 | 1885 | 129 | Orange | 133 |
| HS9 | 1880 | 6079 | Red | 138 |
| HS10 | 1875 | 124 | Black | 143 |
| HS11 | 1875 | 124 | Purple | 143 |
| HS12 | 1861 | 114 | Blue | 157 |
| HS13 | 1861 | 114 | Red | 157 |
| HS14 | 1849 | 3044 | Blue | 169 |
| HS15 | 1849 | 3044 | Red | 169 |
| HS16 | 1840 | 3245 | Yellow | 178 |
| HS17 | 1824 | 111 | Green | 194 |
| HS18 | 1824 | 111 | Red | 194 |
| HS19 | 1824 | 110 | Red | 194 |
| HS20 | 1824 | 122 | Red | 194 |
| HS21 | 1824 | 105 | Orange | 194 |
| HS22 | 1824 | 106 | MNC | 194 |
| HS23 | 1823 | 107 | Orange | 195 |
| HS24 | 1823 | 107 | Red | 195 |
| HS25 | 1815 | 100 | Yellow | 203 |
| HS26 | 1815 | 100 | Yellow | 203 |
| HS27 | 1815 | 101 | Blue | 203 |
| HS28 | 1815 | 101 | Red | 203 |
| HS29 | 1815 | 100 | MNC | 203 |
| HS30 | 1815 | 101 | MNC | 203 |
| HS31 | 1813 | 103 | Blue | 205 |
| HS32 | 1813 | 104 | Red | 205 |
| HS33 | 1808 | 118 | Blue | 210 |
| HS34 | 1808 | 117 | Red | 210 |
| HS35 | 1808 | 117 | Red | 210 |
| HS36 | 1808 | 118 | Red | 210 |
| HS37 | 1790 | 109 | Yellow | 228 |
| HS38 | 1790 | 109 | Red | 228 |
| HS39 | 1700 | 116 | Yellow | 318 |
| HS40 | 1700 | 116 | Red | 318 |
| HS41 | 1700 | 99 | MNC | 318 |
| HS42 | 1700 | 116 | MNC | 318 |
| HS43 | 1550 | 97 | Blue | 468 |
| HS44 | 1550 | 97 | MNC | 468 |
| HS45 | 1538 | 3305 | Blue | 480 |
| HS46 | 1538 | 3305 | Blue | 480 |
| HS47 | 1538 | 3305 | Red | 480 |

SI Table 4. Assigned secondary structures (%) of silk protein obtained from the deconvolution of the Amide I and II region (1720-1480 cm^-1^) of the FPA ATR and reflectance µ-FTIR spectra of commercial (“Mod”) and historical silk (“HS2,32,36,38”).

| Protein secondary structure | MOD reflect. | MOD ATR | HS38 reflect. | HS38 ATR | HS2 reflect. | HS2 ATR | HS32 reflect. | HS32 ATR | HS36 reflect. | HS36 ATR |
| --- | --- | --- | --- | --- | --- | --- | --- | --- | --- | --- |
| (Tyr) side chains/aggregated | 8.95 | 4.51 | 7.26 | 10.66 | 16.30 | 4.67 | 20.93 | 3.18 | 29.61 | 2.77 |
| Aggregate β-strand/  β-sheets (weak)a | 5.89 | 0.00 | 14.43 | 0.00 | 7.88 | 0.00 | 0.00 | 0.00 | 0.00 | 0.00 |
| β-sheets (strong)a | 11.21 | 41.59 | 0.48 | 45.17 | 0.00 | 42.15 | 0.00 | 43.35 | 0.00 | 36.97 |
| β-sheets (strong)b | 2.22 | 0.00 | 5.15 | 0.00 | 1.97 | 0.00 | 14.60 | 0.00 | 12.17 | 0.00 |
| Random coils/extended chains | 18.65 | 0.00 | 24.92 | 0.00 | 27.75 | 0.00 | 2.96 | 0.00 | 10.86 | 0.00 |
| Random coils | 7.72 | 38.78 | 3.42 | 30.10 | 0.00 | 23.70 | 8.85 | 37.50 | 4.87 | 49.69 |
| α-helices | 8.24 | 0.00 | 7.58 | 0.00 | 3.14 | 0.00 | 9.22 | 0.00 | 10.91 | 0.00 |
| β turns 1663-1670 | 9.84 | 1.57 | 9.09 | 0.00 | 5.81 | 1.07 | 0.00 | 0.00 | 7.68 | 0.00 |
| β turns 1671-1685 | 12.04 | 8.74 | 17.16 | 5.40 | 14.78 | 9.04 | 17.86 | 10.51 | 16.27 | 0.10 |
| β turns 1686-1696 | 6.10 | 0.00 | 3.82 | 1.70 | 14.32 | 7.36 | 12.02 | 0.00 | 7.63 | 0.00 |
| β -sheets (weak)a | 9.13 | 4.81 | 6.67 | 6.98 | 8.05 | 7.20 | 10.60 | 3.56 | 0.00 | 9.11 |
| Oxidation | 0.00 | 0.00 | 0.00 | 0.00 | 0.00 | 4.81 | 2.96 | 1.91 | 0.00 | 1.37 |

For β-sheets: “a”: intermolecular; “b”: intramolecular.


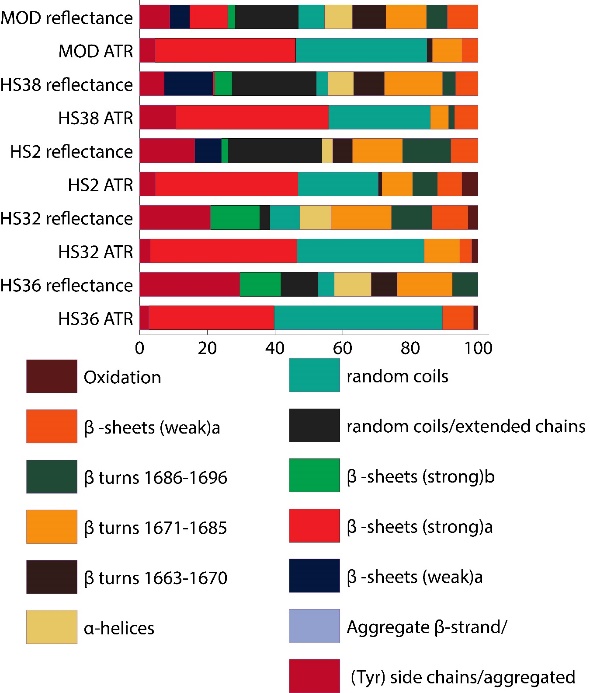


SI Figure 1. Bar plot of the assigned secondary structures (%) of silk protein (also shown in SI Table 4), obtained from the deconvolution of the Amide I and II region (1720-1480 cm^-1^) of the FPA ATR and reflectance µ-FTIR spectra of commercial (“Mod”) and historical silk (“HS2,32,36,38”).


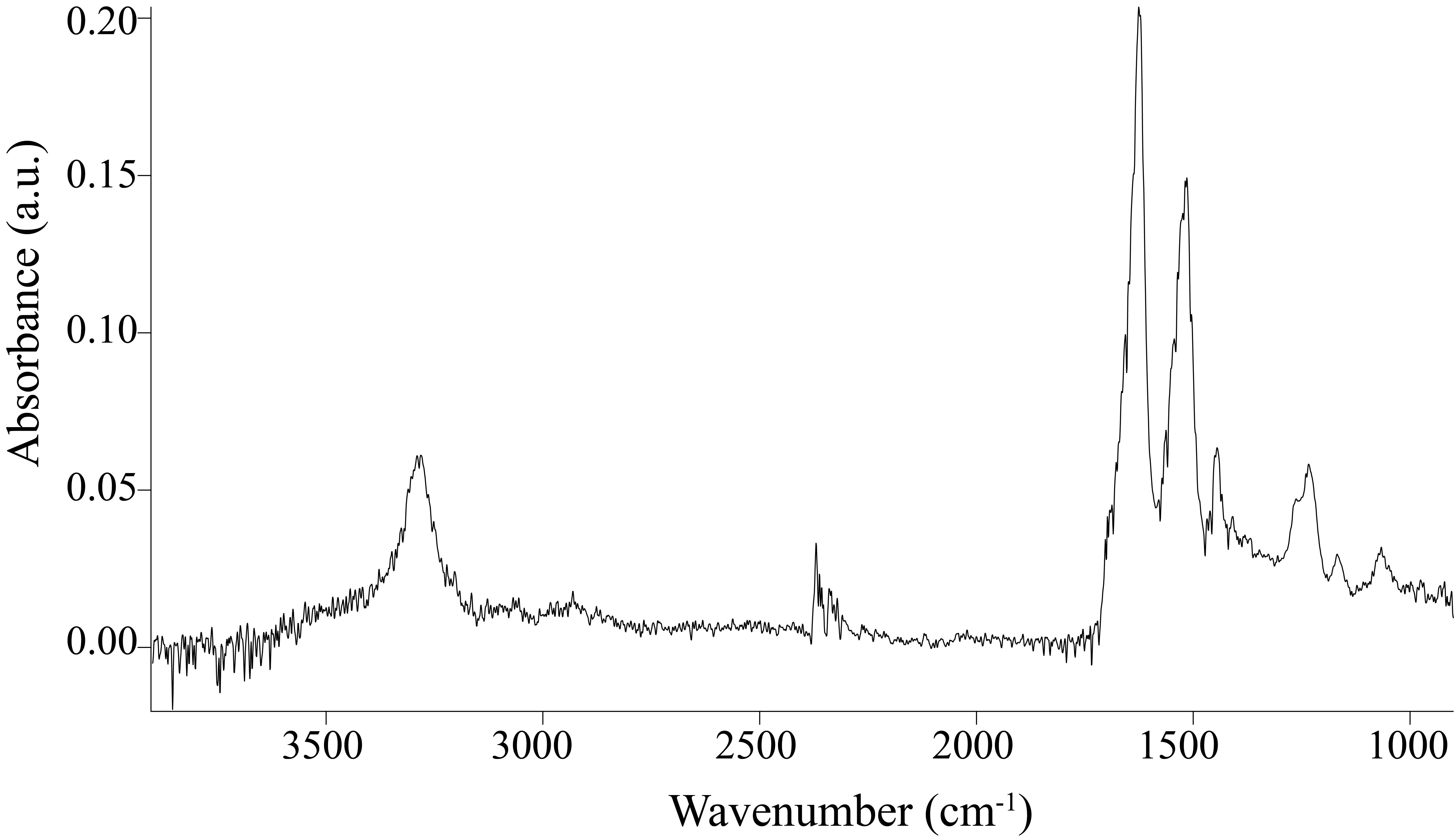

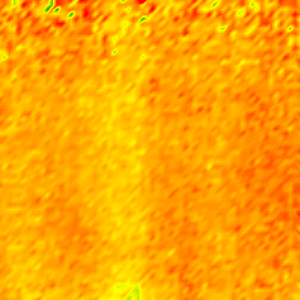

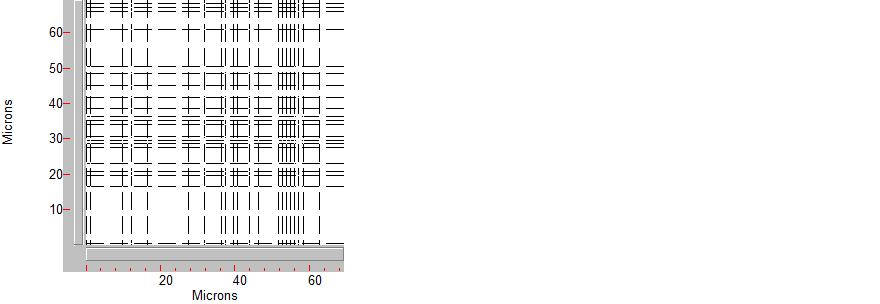


A

B

SI Figure 2. A) ATR 2D FPA map of the intensity of the Amide A band (in the 3440-3160 cm^-1^ range) for the “Mod” silk textile. B) ATR spectrum related to one pixel (1.1 x 1.1 µm^2^) of the map.


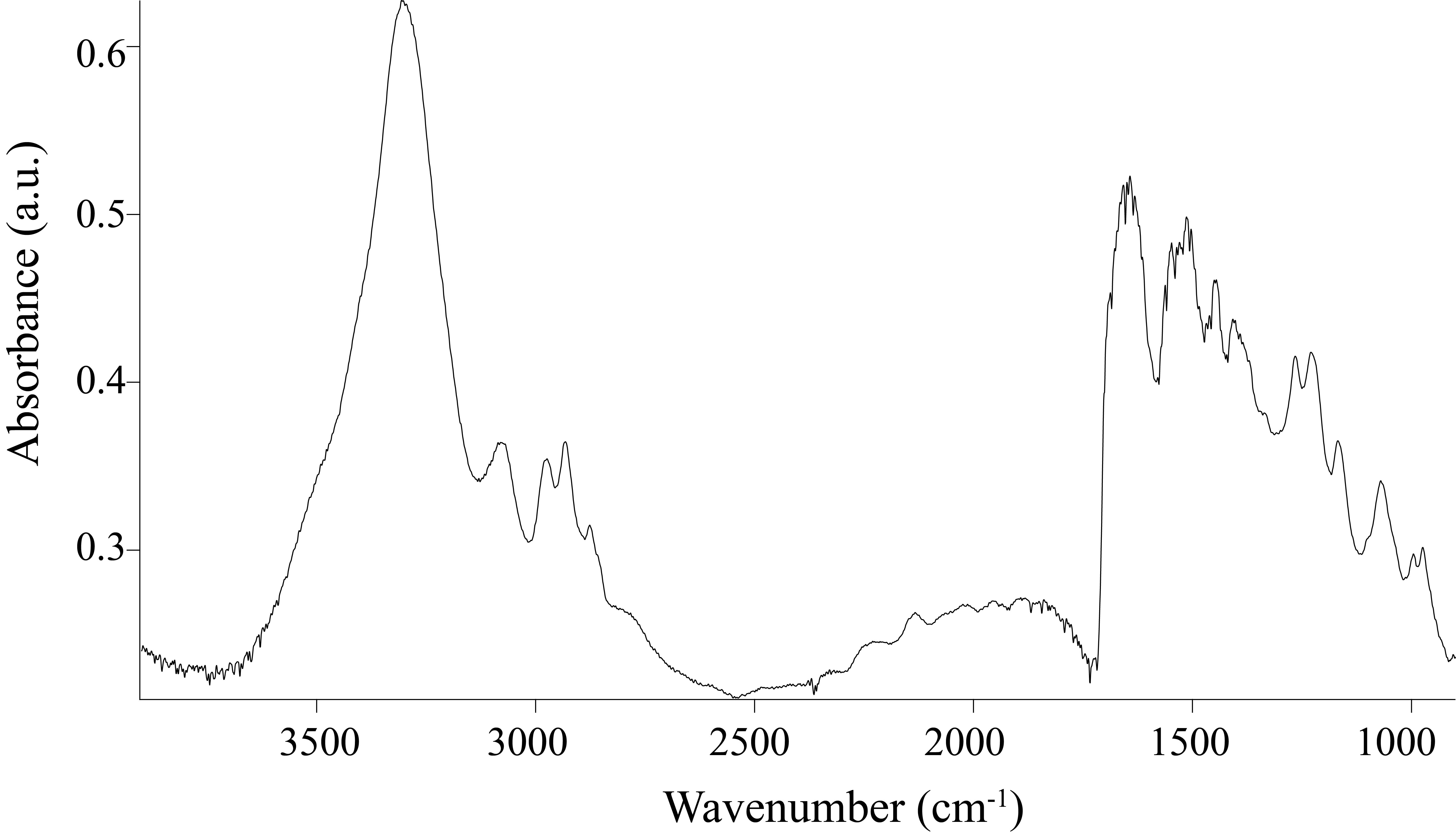

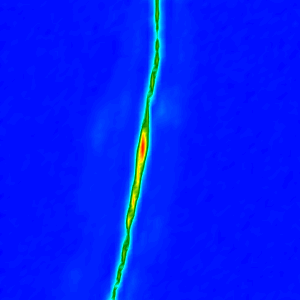

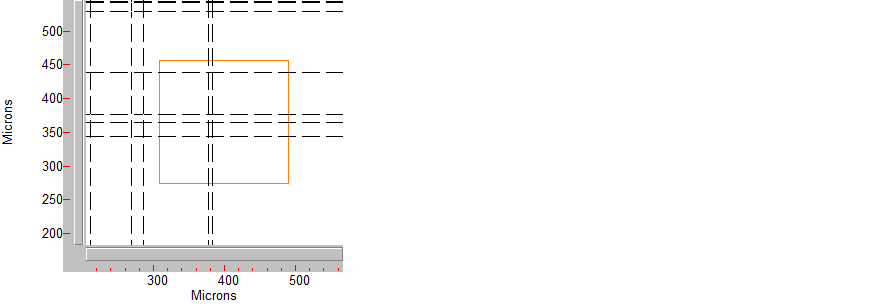


B

A

SI Figure 3. A) Reflectance 2D FPA map of the intensity of the Amide A band (in the 3440-3160 cm^-1^ range) for the “Mod” silk textile. B) Reflectance spectrum related to one pixel (5.5 x 5.5 µm^2^) of the map.


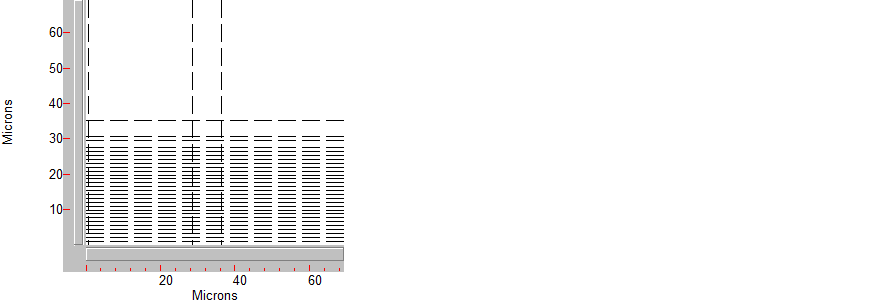

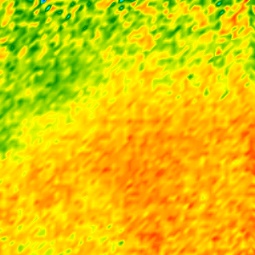

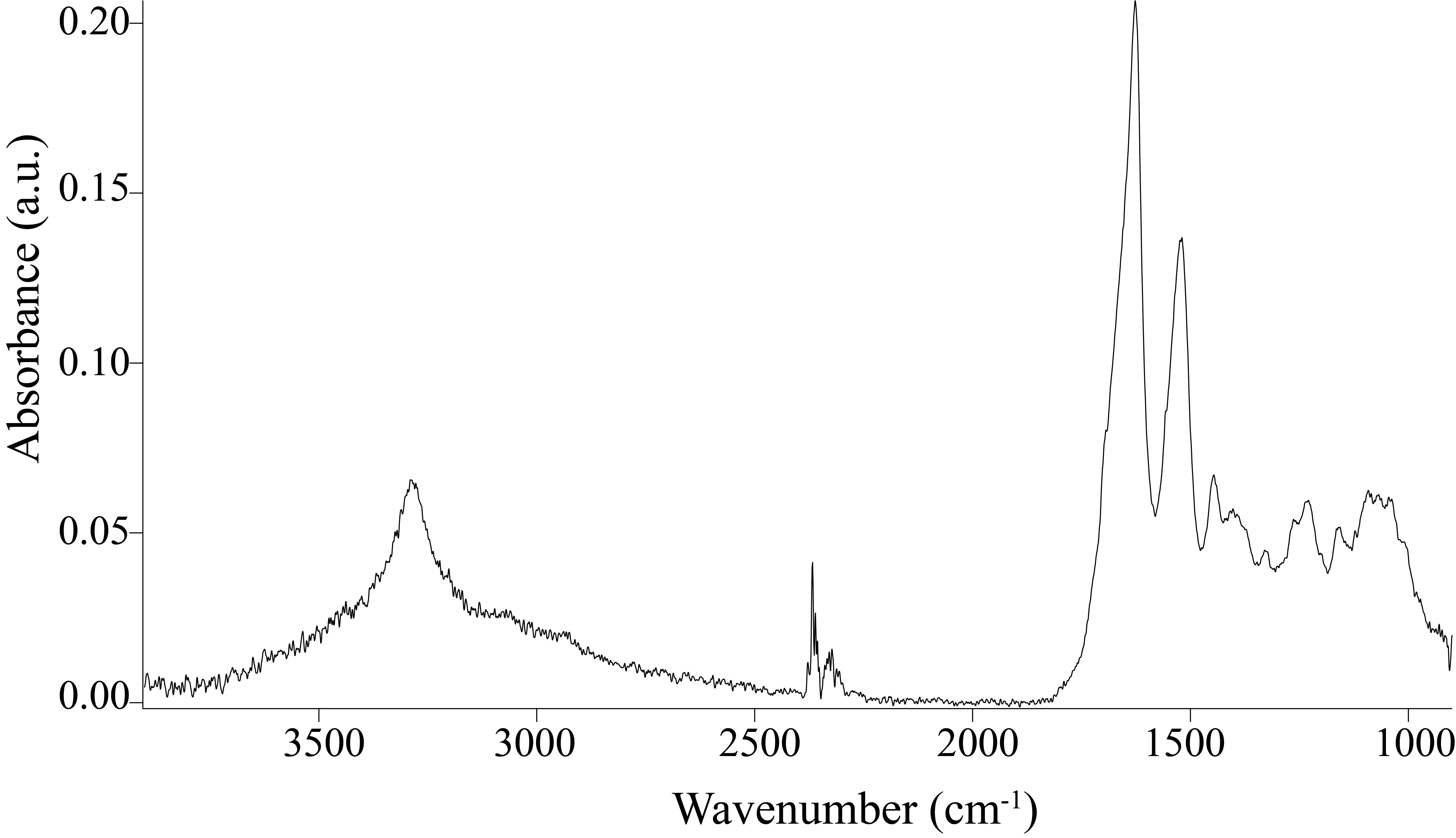


A

B

SI Figure 4. A) ATR 2D FPA map of the intensity of the Amide A band (in the 3440-3160 cm^-1^ range) for the “HS2” silk textile. B) ATR spectrum related to one pixel (1.1 x 1.1 µm^2^) of the map.


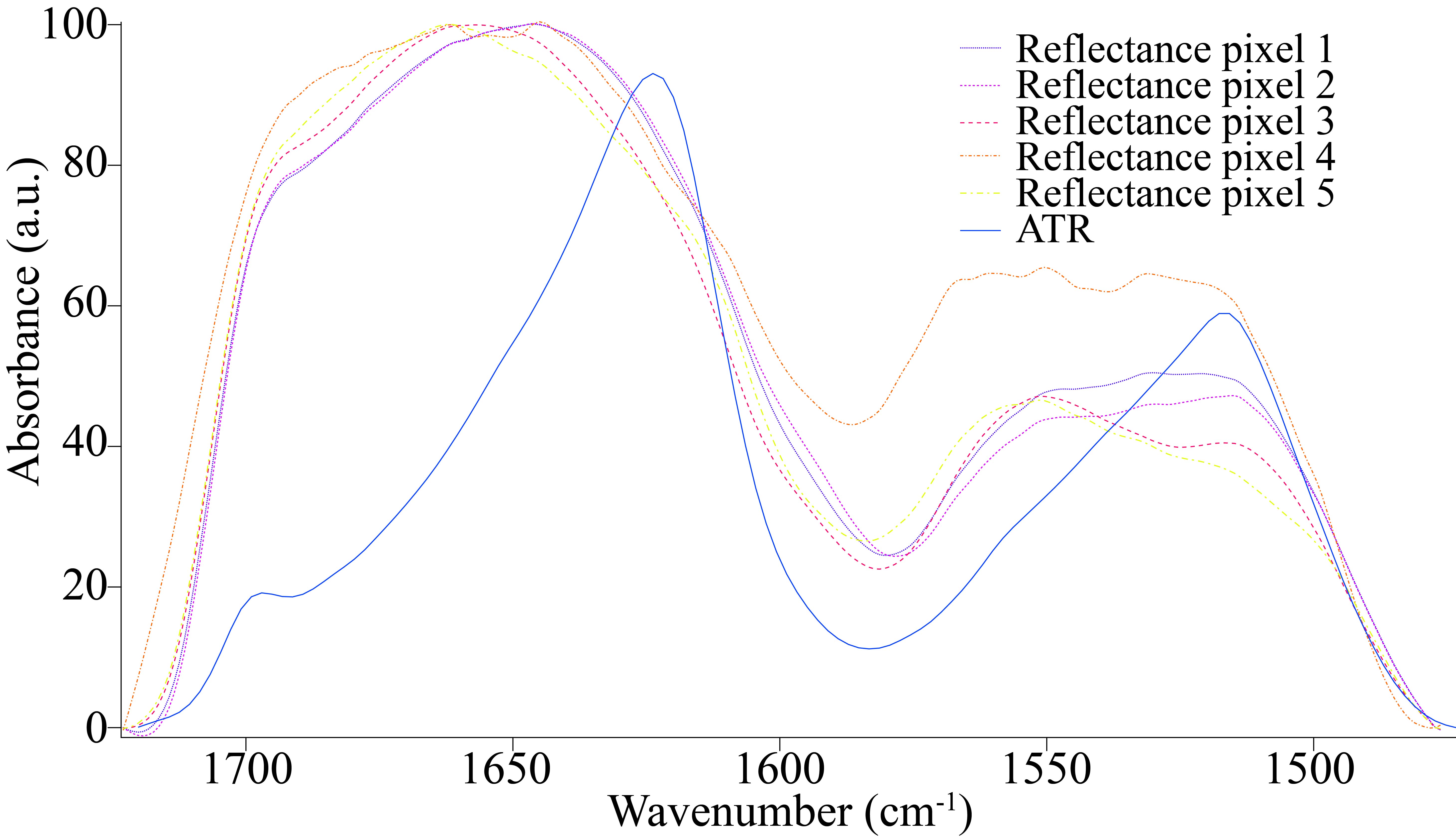


SI Figure 5. Comparison of an ATR spectrum (related to one pixel from the 2D FTIR map shown in Figure SI2B) and five reflectance spectra (related to five different pixels from the 2D FTIR map shown in Figure SI3B) of the “Mod” sample in the Amide I and II region (1750-1450 cm^-1^)


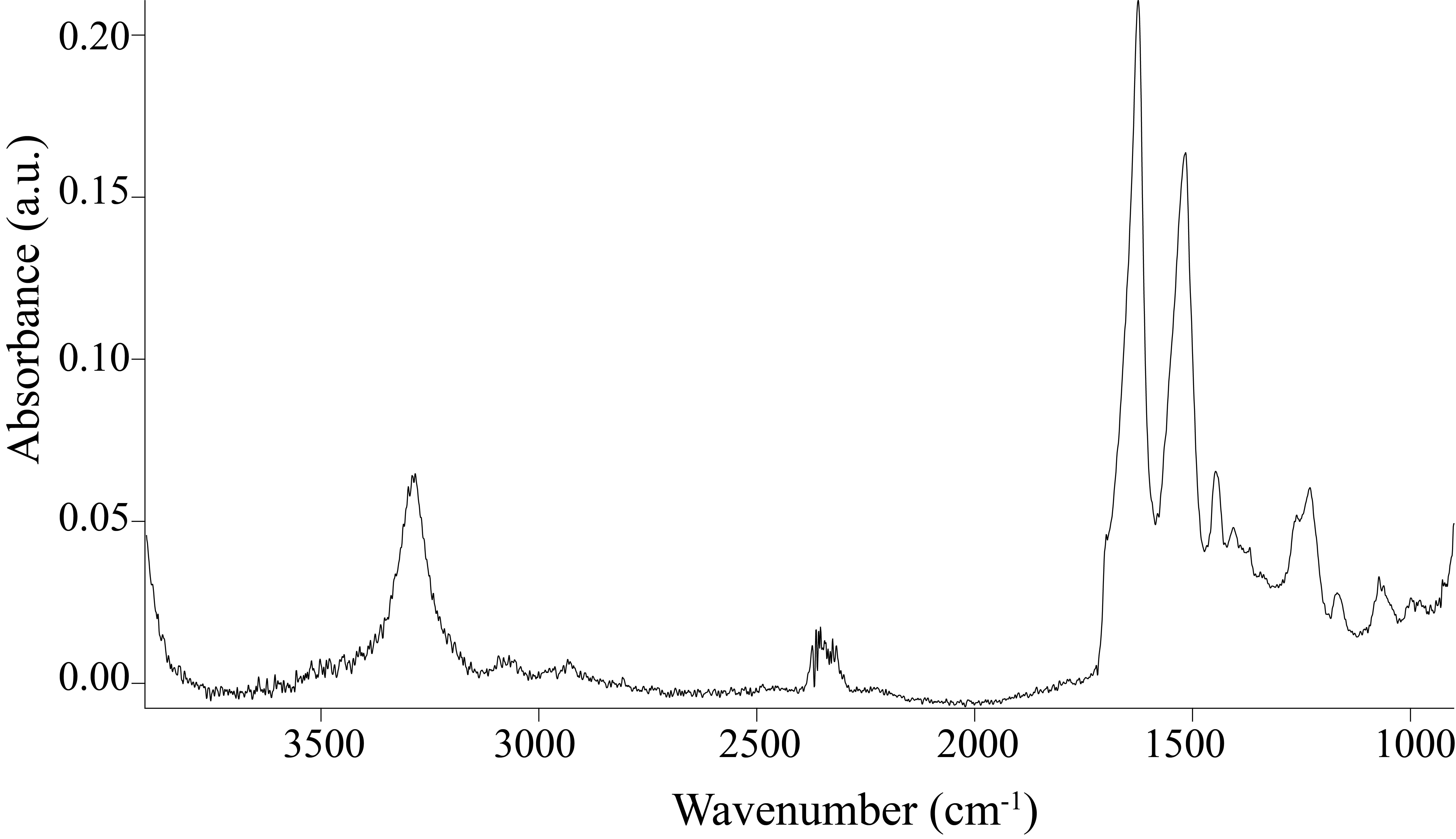


SI Figure 6. ATR Spectra (3900-900 cm^-1^) of the “Mod2” sample.


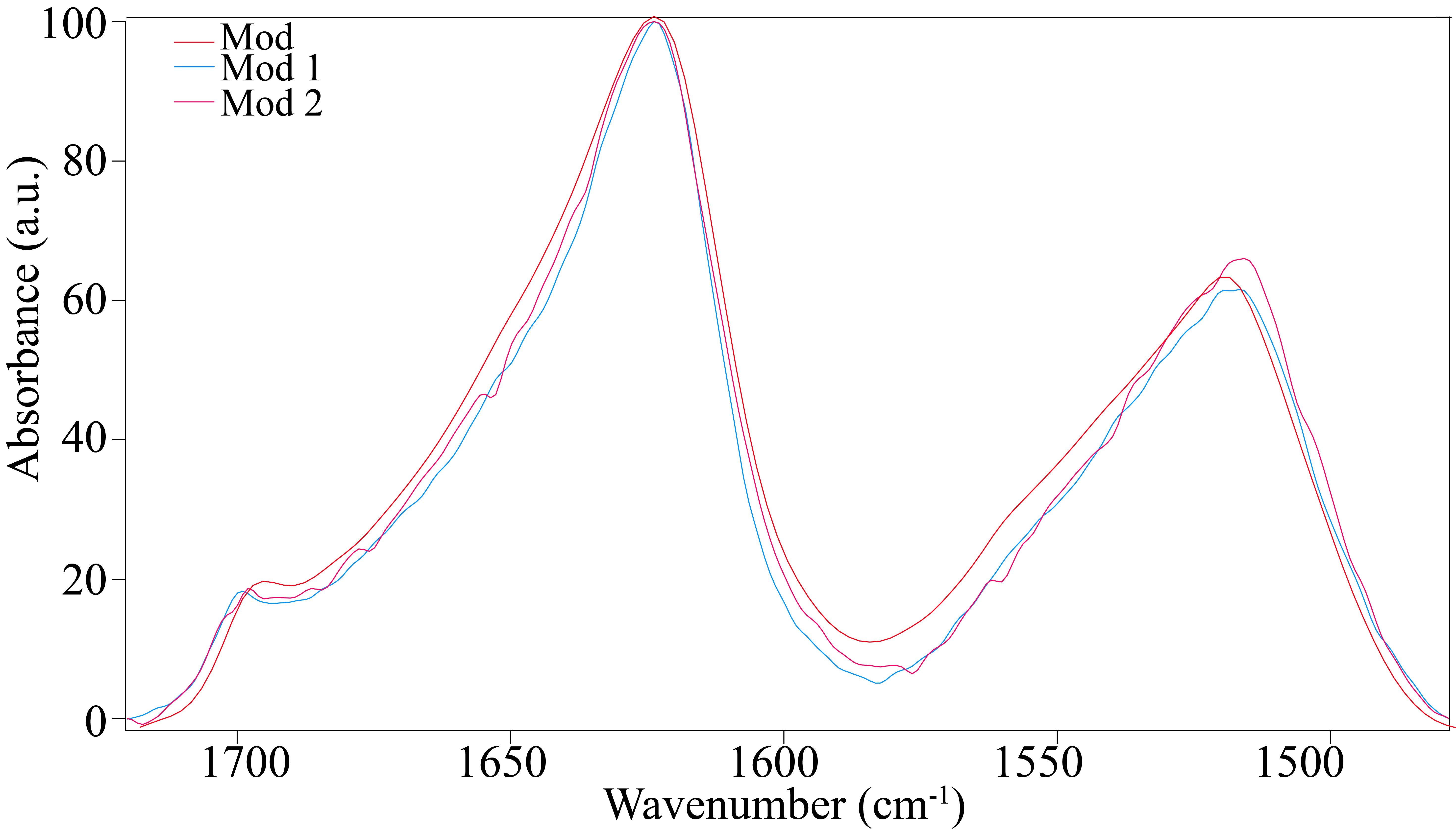


SI Figure 7. Comparison of ATR spectra of the “Mod”, “Mod1” and “Mod2” samples, in the Amide I and II region (1750-1450 cm^-1^).

SI Table 5. Assigned secondary structures (%) of silk protein obtained from the deconvolution of the Amide I and II region (1720-1480 cm^-1^) of the FPA reflectance µ-FTIR spectra of commercial (“Mod”, “Mod1-2”) and historical silk samples (“HS1-47”). (For β-sheets: “a”: intermolecular; “b”: intramolecular.)

| Sample | (Tyr) side chains/ aggregated | Aggregate β-strand/ β -sheets (weak)a | | | β -sheets (strong)a | | β -sheets (strong)b | Random coils/ extended chains | | Random coils | α-helices | | β -Turns 1663-1670 | | β -Turns 1671-1685 | | β -Turns 1686-1696 | | β -sheets (weak)a | | Oxidation |
| --- | --- | --- | --- | --- | --- | --- | --- | --- | --- | --- | --- | --- | --- | --- | --- | --- | --- | --- | --- | --- | --- |
| Mod | 8.95 | | 5.89 | 11.21 | | 2.22 | | | 18.65 | 7.72 | | 8.24 | | 9.84 | 12.04 | 6.10 | | 9.13 | | 0.00 | |
| Mod1 | 13.73 | | 0.00 | 5.18 | | 23.11 | | | 13.07 | 2.37 | | 6.70 | | 3.67 | 19.95 | 4.70 | | 5.62 | | 1.89 | |
| Mod2 | 11.06 | | 2.64 | 4.99 | | 10.53 | | | 12.26 | 9.51 | | 12.04 | | 2.62 | 19.65 | 6.54 | | 4.43 | | 3.17 | |
| HS1 | 16.45 | | 5.10 | 3.02 | | 15.49 | | | 6.82 | 7.98 | | 5.87 | | 13.17 | 16.42 | 4.46 | | 5.22 | | 0.00 | |
| HS2 | 16.30 | | 7.88 | 0.00 | | 1.97 | | | 27.75 | 0.00 | | 3.14 | | 5.81 | 14.78 | 14.32 | | 8.05 | | 0.00 | |
| HS3 | 20.19 | | 12.08 | 3.29 | | 5.62 | | | 5.95 | 13.50 | | 6.53 | | 8.41 | 16.24 | 4.63 | | 3.57 | | 0.00 | |
| HS4 | 26.73 | | 0.00 | 1.40 | | 10.53 | | | 11.83 | 2.35 | | 5.52 | | 5.41 | 27.37 | 8.68 | | 0.20 | | 0.00 | |
| HS5 | 20.90 | | 0.00 | 0.00 | | 5.77 | | | 7.42 | 7.77 | | 12.51 | | 4.11 | 11.85 | 0.00 | | 5.78 | | 23.90 | |
| HS6 | 18.79 | | 0.00 | 2.77 | | 5.08 | | | 22.60 | 2.19 | | 1.82 | | 21.23 | 1.02 | 16.77 | | 7.73 | | 0.00 | |
| HS7 | 25.45 | | 0.00 | 2.37 | | 5.37 | | | 15.24 | 13.40 | | 3.72 | | 9.43 | 12.58 | 7.95 | | 4.50 | | 0.00 | |
| HS8 | 17.70 | | 1.74 | 5.20 | | 11.96 | | | 2.07 | 11.82 | | 5.79 | | 16.39 | 15.10 | 4.85 | | 7.38 | | 0.00 | |
| HS9 | 24.91 | | 0.00 | 3.22 | | 7.73 | | | 2.22 | 15.26 | | 4.44 | | 11.92 | 20.23 | 10.07 | | 0.00 | | 0.00 | |
| HS10 | 18.51 | | 0.00 | 3.88 | | 9.35 | | | 7.62 | 3.26 | | 11.55 | | 4.61 | 12.45 | 10.49 | | 13.28 | | 5.00 | |
| HS11 | 22.23 | | 0.00 | 0.00 | | 4.00 | | | 21.87 | 5.85 | | 1.38 | | 26.30 | 11.57 | 5.95 | | 0.84 | | 0.00 | |
| HS12 | 12.53 | | 13.55 | 0.00 | | 11.58 | | | 14.88 | 4.46 | | 5.35 | | 16.16 | 7.11 | 10.43 | | 3.95 | | 0.00 | |
| HS13 | 25.43 | | 2.22 | 16.27 | | 9.00 | | | 4.54 | 7.20 | | 16.22 | | 0.00 | 13.17 | 5.94 | | 0.00 | | 0.00 | |
| HS14 | 16.81 | | 0.00 | 0.00 | | 18.62 | | | 7.98 | 0.93 | | 11.98 | | 8.01 | 14.49 | 13.30 | | 7.02 | | 0.86 | |
| HS15 | 11.80 | | 9.96 | 4.23 | | 13.05 | | | 0.15 | 15.88 | | 12.99 | | 2.43 | 18.43 | 4.90 | | 6.18 | | 0.00 | |
| HS16 | 13.20 | | 10.03 | 0.00 | | 18.93 | | | 5.23 | 4.77 | | 19.17 | | 2.68 | 16.39 | 8.46 | | 1.16 | | 0.00 | |
| HS17 | 16.25 | | 0.00 | 8.41 | | 5.12 | | | 13.28 | 9.28 | | 3.17 | | 6.12 | 24.38 | 3.53 | | 6.61 | | 3.84 | |
| HS18 | 22.96 | | 0.94 | 12.11 | | 6.26 | | | 23.73 | 0.00 | | 11.28 | | 2.15 | 17.93 | 2.64 | | 0.00 | | 0.00 | |
| HS19 | 16.89 | | 0.00 | 7.75 | | 9.75 | | | 0.00 | 21.29 | | 0.00 | | 0.00 | 15.50 | 13.52 | | 9.77 | | 5.53 | |
| HS20 | 15.05 | | 0.82 | 14.64 | | 6.86 | | | 8.87 | 21.65 | | 0.86 | | 6.15 | 14.20 | 8.58 | | 2.31 | | 0.00 | |
| HS21 | 25.98 | | 0.00 | 2.93 | | 6.49 | | | 12.08 | 13.17 | | 0.00 | | 8.85 | 14.47 | 10.73 | | 5.30 | | 0.00 | |
| HS22 | 24.62 | | 6.51 | 0.00 | | 28.99 | | | 6.01 | 5.29 | | 6.01 | | 5.97 | 9.03 | 5.14 | | 2.42 | | 0.00 | |
| HS23 | 11.48 | | 0.00 | 6.60 | | 17.87 | | | 3.06 | 12.29 | | 13.01 | | 1.54 | 21.59 | 5.19 | | 7.39 | | 0.00 | |
| HS24 | 14.15 | | 5.69 | 3.04 | | 17.39 | | | 7.10 | 9.12 | | 0.00 | | 3.19 | 27.25 | 12.49 | | 0.57 | | 0.00 | |
| HS25 | 12.09 | | 12.57 | 0.52 | | 8.08 | | | 21.79 | 6.33 | | 10.28 | | 0.00 | 21.44 | 6.91 | | 0.00 | | 0.00 | |
| HS26 | 7.36 | | 8.43 | 6.46 | | 6.10 | | | 3.74 | 21.58 | | 1.44 | | 4.89 | 20.10 | 3.22 | | 10.19 | | 6.48 | |
| HS27 | 16.83 | | 0.00 | 5.01 | | 10.36 | | | 12.28 | 6.69 | | 3.76 | | 14.39 | 16.75 | 0.00 | | 10.00 | | 3.91 | |
| HS28 | 10.50 | | 11.41 | 2.99 | | 4.69 | | | 5.20 | 12.17 | | 7.60 | | 3.08 | 16.71 | 5.28 | | 17.66 | | 2.72 | |
| HS29 | 17.69 | | 1.88 | 3.64 | | 11.78 | | | 8.16 | 13.58 | | 0.00 | | 25.08 | 4.95 | 8.68 | | 4.56 | | 0.00 | |
| HS30 | 16.85 | | 3.75 | 0.00 | | 15.10 | | | 16.78 | 5.27 | | 7.02 | | 13.60 | 7.08 | 8.74 | | 5.81 | | 0.00 | |
| HS31 | 14.14 | | 3.64 | 9.94 | | 0.00 | | | 11.26 | 13.65 | | 0.00 | | 18.66 | 9.54 | 10.77 | | 6.76 | | 1.64 | |
| HS32 | 20.93 | | 0.00 | 0.00 | | 14.60 | | | 2.96 | 8.85 | | 9.22 | | 0.00 | 17.86 | 12.02 | | 10.60 | | 2.96 | |
| HS33 | 15.19 | | 5.71 | 0.00 | | 13.55 | | | 6.62 | 17.17 | | 7.25 | | 6.78 | 13.49 | 11.26 | | 2.98 | | 0.00 | |
| HS34 | 15.67 | | 0.00 | 11.23 | | 6.19 | | | 13.36 | 2.01 | | 5.24 | | 12.97 | 13.19 | 9.15 | | 8.99 | | 2.00 | |
| HS35 | 19.94 | | 0.00 | 8.65 | | 5.54 | | | 16.26 | 0.00 | | 8.05 | | 5.07 | 27.22 | 6.28 | | 2.98 | | 0.00 | |
| HS36 | 29.61 | | 0.00 | 0.00 | | 12.17 | | | 10.86 | 4.87 | | 10.91 | | 7.68 | 16.27 | 7.63 | | 0.00 | | 0.00 | |
| HS37 | 7.26 | | 14.43 | 0.48 | | 5.15 | | | 24.92 | 3.42 | | 7.58 | | 9.09 | 17.16 | 3.82 | | 6.67 | | 0.00 | |
| HS38 | 13.22 | | 5.30 | 4.14 | | 7.85 | | | 9.69 | 14.88 | | 6.10 | | 6.92 | 18.46 | 7.00 | | 5.91 | | 0.53 | |
| HS39 | 20.15 | | 0.00 | 0.00 | | 4.63 | | | 32.74 | 7.03 | | 2.89 | | 0.00 | 22.22 | 8.67 | | 1.66 | | 0.00 | |
| HS40 | 12.24 | | 17.77 | 2.40 | | 5.32 | | | 14.07 | 4.89 | | 14.17 | | 4.66 | 12.56 | 7.88 | | 3.65 | | 0.40 | |
| HS41 | 15.49 | | 4.32 | 7.42 | | 11.48 | | | 12.64 | 4.13 | | 16.16 | | 0.94 | 18.66 | 6.65 | | 2.11 | | 0.00 | |
| HS42 | 22.11 | | 0.00 | 3.18 | | 6.29 | | | 16.53 | 11.57 | | 0.00 | | 9.36 | 17.60 | 4.72 | | 8.64 | | 0.00 | |
| HS43 | 13.53 | | 0.00 | 11.49 | | 2.51 | | | 21.63 | 3.65 | | 12.19 | | 6.14 | 7.17 | 16.42 | | 5.14 | | 0.13 | |
| HS44 | 11.85 | | 0.00 | 21.56 | | 14.61 | | | 3.19 | 7.07 | | 0.00 | | 22.34 | 6.92 | 10.66 | | 1.79 | | 0.00 | |
| HS45 | 23.08 | | 0.00 | 12.78 | | 7.81 | | | 21.39 | 0.00 | | 5.98 | | 8.70 | 6.57 | 10.08 | | 3.61 | | 0.00 | |
| HS46 | 19.00 | | 5.29 | 0.00 | | 11.02 | | | 4.09 | 11.99 | | 3.64 | | 10.05 | 20.81 | 0.00 | | 13.78 | | 0.33 | |
| HS47 | 16.62 | | 7.34 | 2.29 | | 12.36 | | | 8.51 | 9.70 | | 7.07 | | 2.18 | 19.56 | 8.93 | | 5.45 | | 0.00 | |


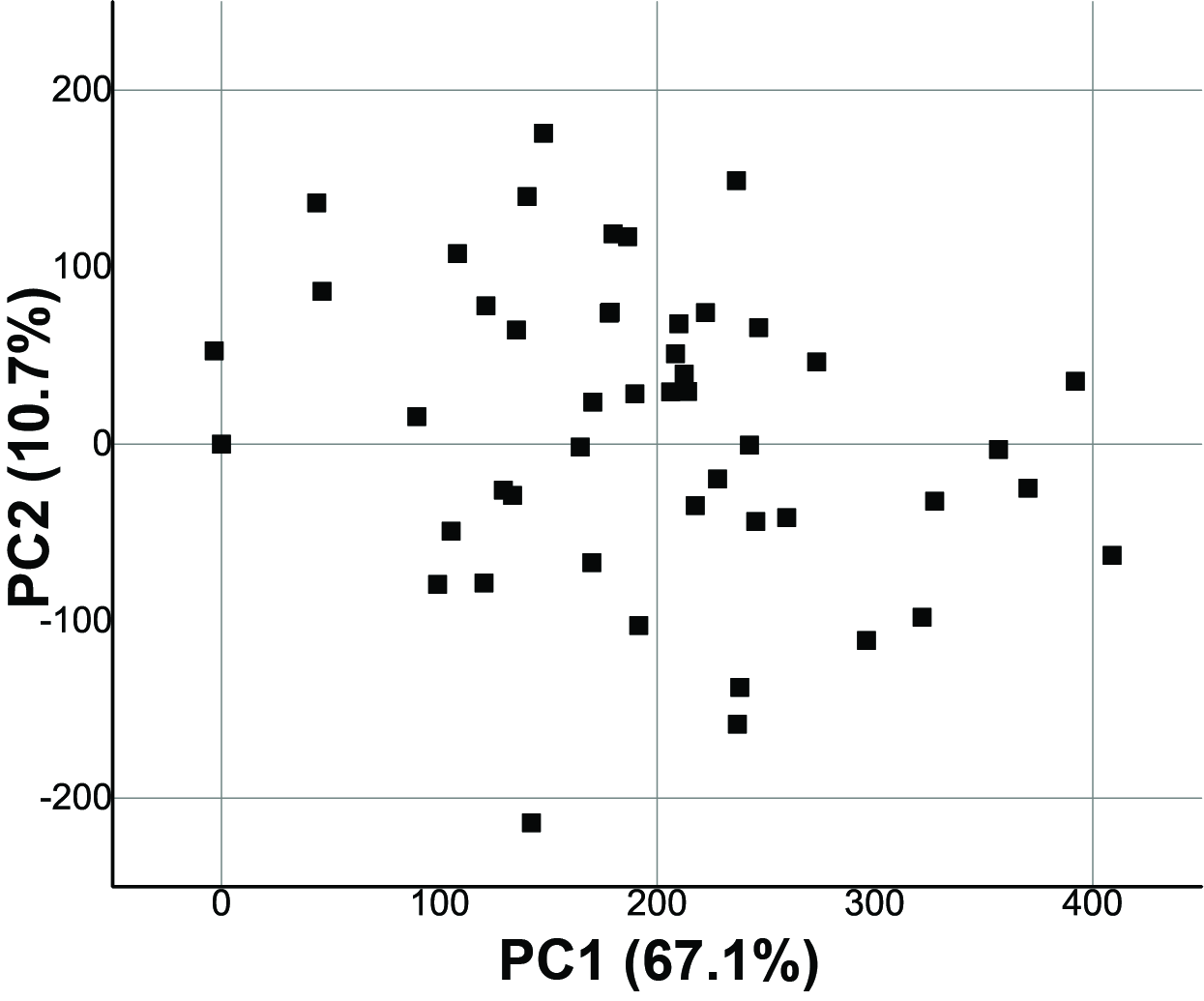


SI 8. Score plot obtained from PCA considering as variables the relative amounts (%) of different secondary structures (from FTIR measurements and deconvolution fitting process) for a set of 50 silk samples (Mod, Mod1-2 and HS1-47), and the age of the samples.

SI Table 6. Dyes identification after FORS analysis of the historical samples HS1-HS47. MNC: Fiber not colored, originally located inside a metal thread in the artifact; --: Not measured

| Sample | Color | Age | L* | A* | B* | Dye assignment |
| --- | --- | --- | --- | --- | --- | --- |
| HS1 | Red | 89 | 44.96 | -1.117 | -6.715 | Indigo |
| HS2 | Red | 117 | 40.25 | 23.461 | 5.64 | Madder |
| HS3 | Red | 117 | 39.14 | 19.692 | 2.09 | Cochineal |
| HS4 | Yellow | 118 | 49.96 | -0.517 | 18.785 | Natural yellow dye |
| HS5 | Red | 118 | 28.63 | 31.441 | 6.033 | Brazilwood |
| HS6 | Red | 119 | 48.65 | 6.451 | -2.834 | Brazilwood |
| HS7 | Yellow | 133 | 46.35 | 1.322 | 7.406 | Natural yellow dye |
| HS8 | Orange | 133 | 55.1 | 8.256 | 2.827 | Brazilwood |
| HS9 | Red | 138 | -- | -- | -- | -- |
| HS10 | Black | 143 | 49.78 | -1.437 | -2.544 | -- |
| HS11 | Purple | 143 | 22.55 | 10.652 | -7.835 | Indigo+Cochineal |
| HS12 | Blue | 157 | 24.76 | 0.348 | -16.238 | Prussian Blue |
| HS13 | Red | 157 | 30.63 | 29.229 | 7.269 | Brazilwood |
| HS14 | Blue | 169 | 57.02 | -3.145 | -8.654 | Prussian Blue |
| HS15 | Red | 169 | 31.97 | 28.99 | 6.954 | Brazilwood |
| HS16 | Yellow | 178 | 70.06 | -1.521 | 4.817 | Natural yellow dye |
| HS17 | Green | 194 | 62.95 | -2.43 | -3.348 | Indigo+ yellow dye |
| HS18 | Red | 194 | 34.11 | 15.054 | 11.863 | Madder +Al |
| HS19 | Red | 194 | 48.62 | 8.698 | -1.45 | Cochineal |
| HS20 | Red | 194 | 30.93 | 34.314 | 10.219 | Brazil wood |
| HS21 | Orange | 194 | 28.35 | 16.746 | -1.584 | Cochineal+ yellow dye |
| HS22 | MNC | 194 | -- | -- | -- | -- |
| HS23 | Orange | 195 | -- | -- | -- | -- |
| HS24 | Red | 195 | 33.46 | 19.052 | 4.412 | Cochineal |
| HS25 | Yellow | 203 | 51.71 | -0.642 | 6.447 | Natural yellow dye |
| HS26 | Yellow | 203 | 51.71 | -0.642 | 6.447 | Natural yellow dye |
| HS27 | Blue | 203 | -- | -- | -- | -- |
| HS28 | Red | 203 | 34.34 | 13.051 | 4.337 | Cochineal |
| HS29 | MNC | 203 | -- | -- | -- | -- |
| HS30 | MNC | 203 | -- | -- | -- | -- |
| HS31 | Blue | 205 | -- | -- | -- | -- |
| HS32 | Red | 205 | 33.73 | 25.086 | -0.536 | Cochineal |
| HS33 | Blue | 210 | 70.89 | -3.117 | -4.992 | Indigo |
| HS34 | Red | 210 | 57.09 | -1.568 | 8.015 | black |
| HS35 | Red | 210 | 34.46 | 8.188 | -0.199 | black |
| HS36 | Red | 210 | 31.79 | 21.313 | 4.063 | Cochineal |
| HS37 | Yellow | 228 | 58.02 | -0.846 | 6.247 | Natural yellow dye |
| HS38 | Red | 228 | 33.73 | 20.332 | 5.776 | Cochineal |
| HS39 | Yellow | 318 | 59.26 | -2.429 | 4.836 | Natural yellow dye |
| HS40 | Red | 318 | 37.28 | 16.119 | 4.995 | Cochineal |
| HS41 | MNC | 318 | -- | -- | -- | -- |
| HS42 | MNC | 318 | -- | -- | -- | -- |
| HS43 | Blue | 468 | 44.11 | -6.457 | -11.464 | Indigo |
| HS44 | MNC | 468 | -- | -- | -- | -- |
| HS45 | Blue | 480 | 51.51 | -3.135 | -0.492 | Indigo |
| HS46 | Blue | 480 | 46.7 | -4.982 | -3.663 | Indigo |
| HS47 | Red | 480 | 35.99 | 30.188 | 4.488 | Cochineal |


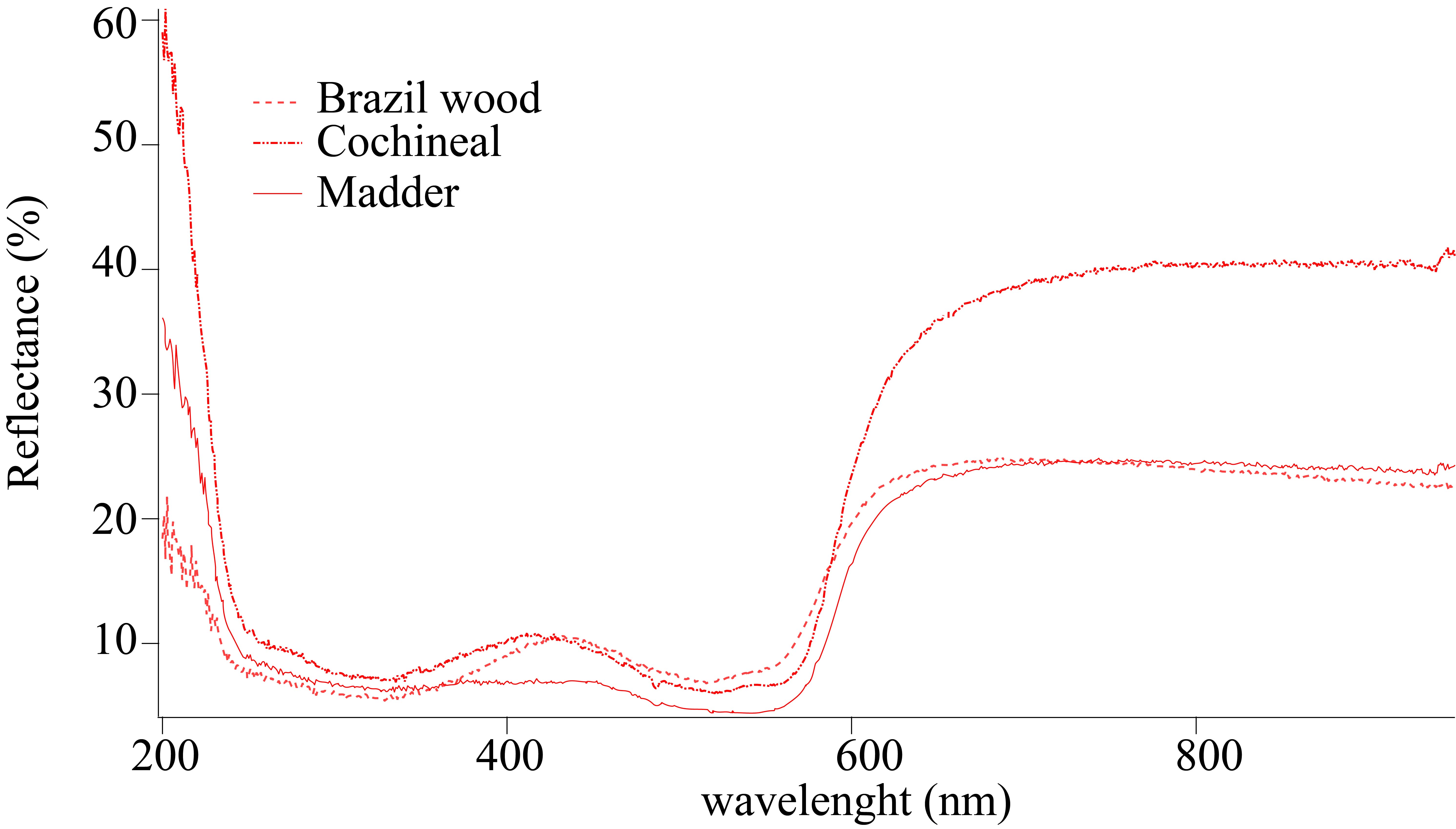


SI Figure 9. Example of FORS Spectra of red dyes present on the silk textiles. The spectra match with reference spectra of, respectively, Cochineal, Brazil wood, and red madder, reported in the literature^1–3^


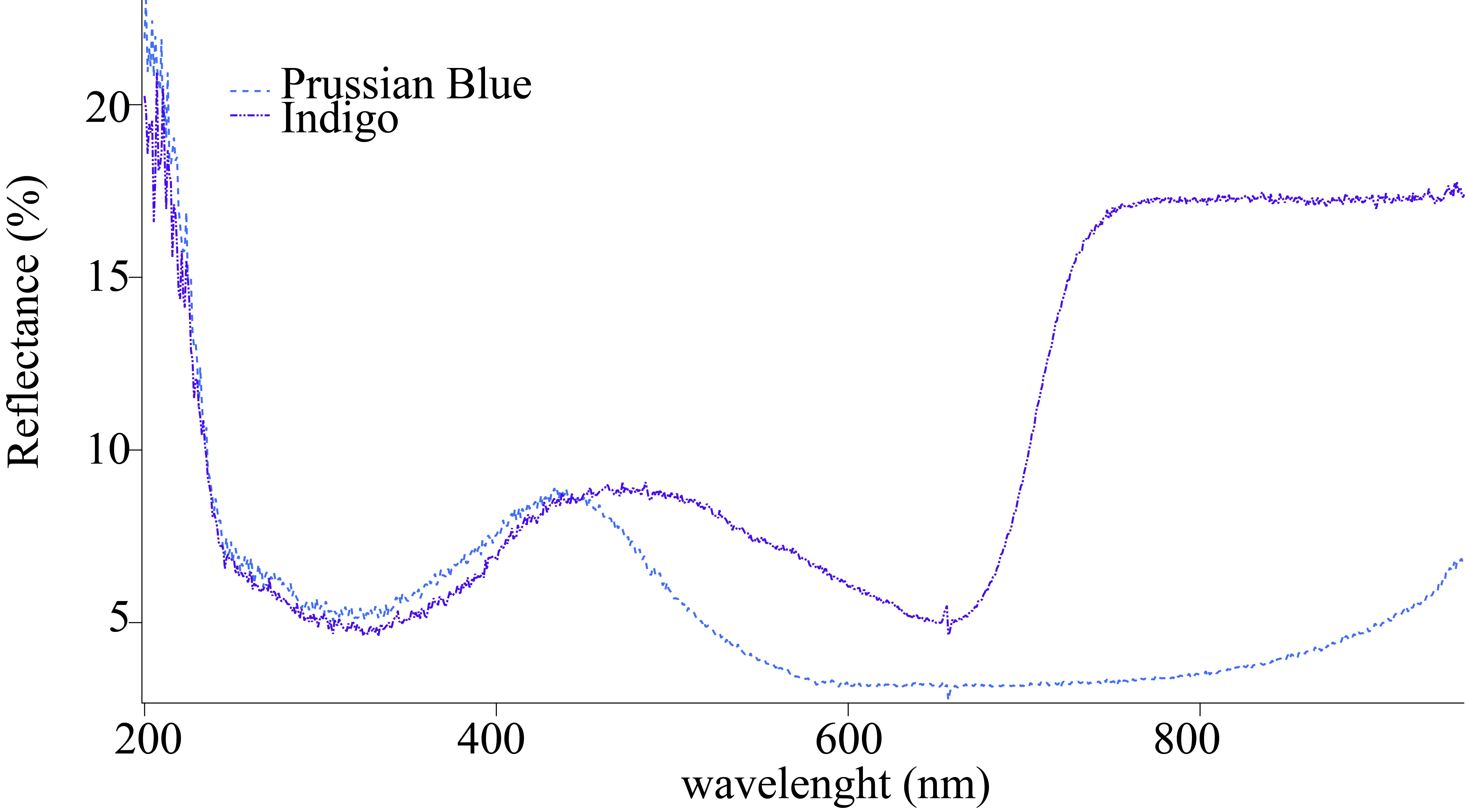


SI Figure 10. Example of FORS Spectra of blue dyes present on the silk textiles. The spectra match with reference spectra of, respectively, Indigo and Prussian blue, reported in the literature^1,4^


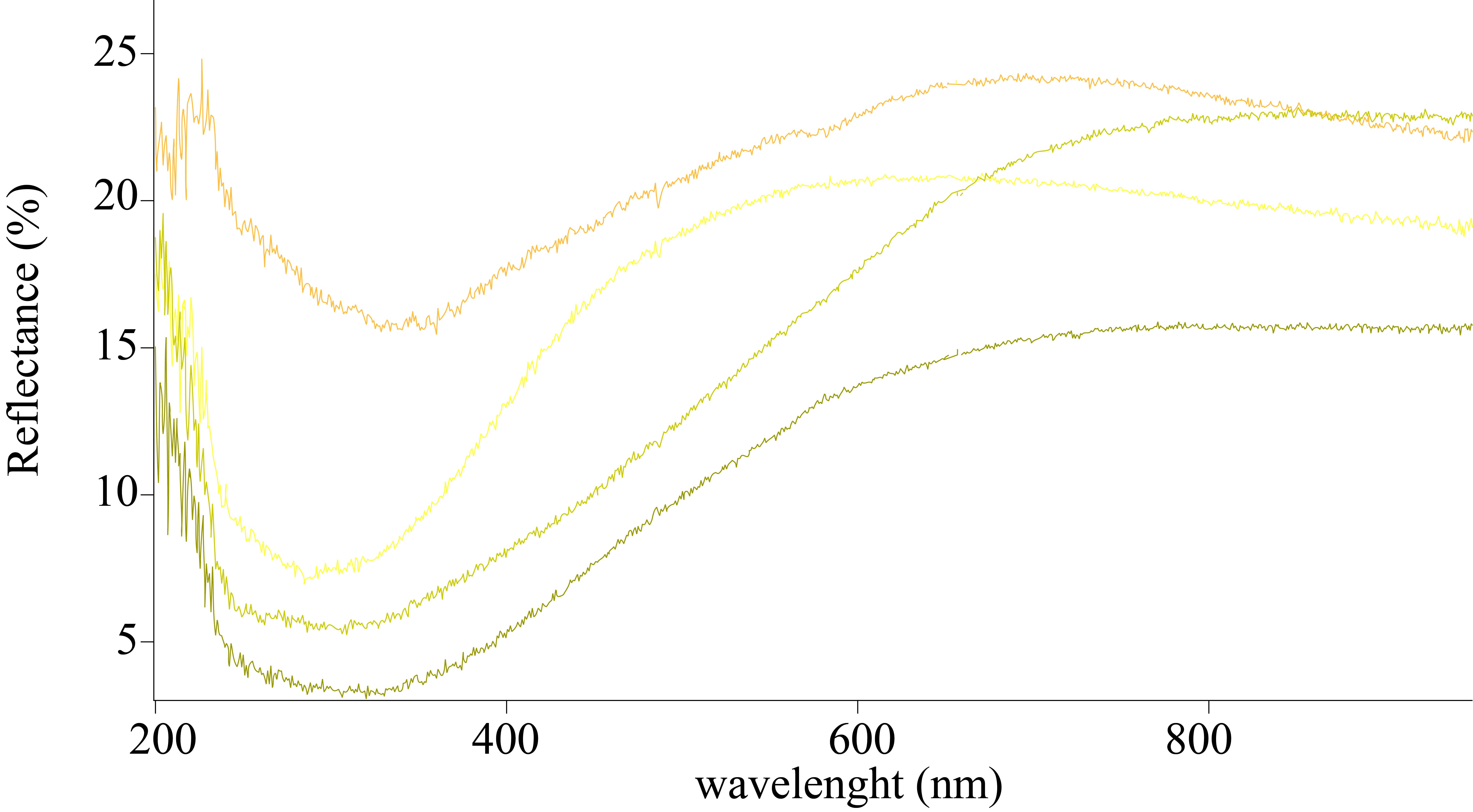


SI Figure 11. Example of FORS Spectra of yellow dyes present on the silk textiles. Even though it was not possible to unequivocally identify the yellow dyes by FORS, their spectra resemble those of some natural dyes commonly and historically used, such as hydroxy and methoxy derivatives of flavones and isoflavones, dihydropyrans, anthocyananidins and carotenoids ^2,5,6^


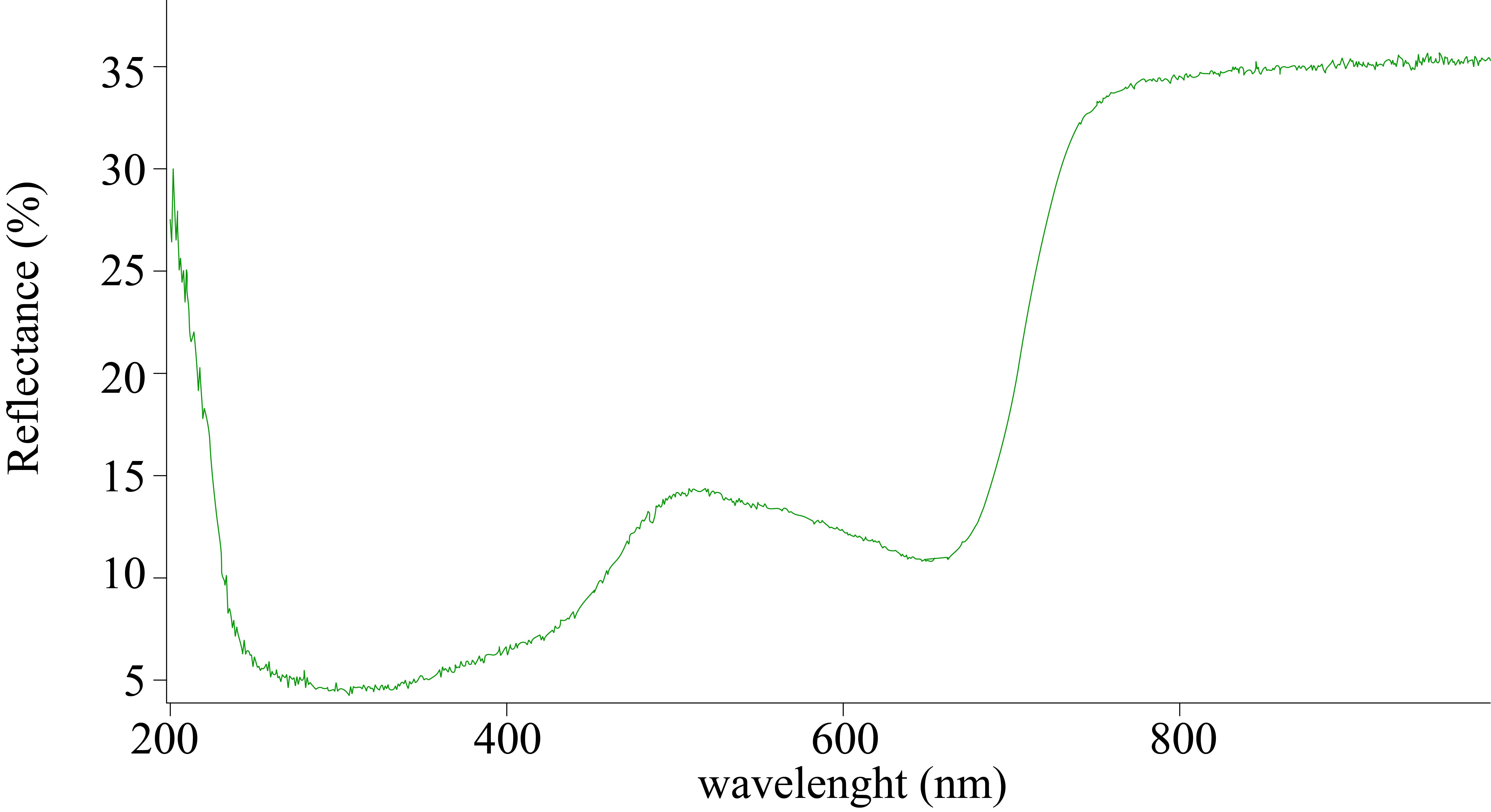


SI Figure 12. Example of FORS spectra of green dye identified on the silk textiles: The color is achieved through the combination of two natural dyes (a yellow natural dye plus indigo)^7^

1. Leona, M. & Winter, J. Fiber Optics Reflectance Spectroscopy: A Unique Tool for the Investigation of Japanese Paintings. *Stud. Conserv.* **46**, 153–162 (2001).

2. Angelini, L. G. *et al.* Characterization of Traditional Dyes of the Mediterranean Area by Non-Invasive Uv-Vis-Nir Reflectance Spectroscopy. *Stud. Conserv.* **55**, 184–189 (2010).

3. Maynez-Rojas, M. A., Casanova-González, E. & Ruvalcaba-Sil, J. L. Identification of natural red and purple dyes on textiles by Fiber-optics Reflectance Spectroscopy. *Spectrochim. Acta. A. Mol. Biomol. Spectrosc.* **178**, 239–250 (2017).

4. Bacci, M., Magrini, D., Picollo, M. & Vervat, M. A study of the blue colors used by Telemaco Signorini (1835–1901). *J. Cult. Herit.* **10**, 275–280 (2009).

5. Gulmini, M. *et al.* Identification of dyestuffs in historical textiles: Strong and weak points of a non-invasive approach. *Dyes Pigments* **98**, 136–145 (2013).

6. Montagner, C., Bacci, M., Bracci, S., Freeman, R. & Picollo, M. Library of UV–Vis–NIR reflectance spectra of modern organic dyes from historic pattern-card coloured papers. *Spectrochim. Acta. A. Mol. Biomol. Spectrosc.* **79**, 1669–1680 (2011).

7. de Ferri, L. *et al.* Non-invasive study of natural dyes on historical textiles from the collection of Michelangelo Guggenheim. *Spectrochim. Acta. A. Mol. Biomol. Spectrosc.* **204**, 548–567 (2018).
